# Enhanced upregulation of SIRT1 via pioglitazone and ligustrazine confers protection against ethanol-induced gastric ulcer in rats

**DOI:** 10.1007/s00210-024-03026-6

**Published:** 2024-03-05

**Authors:** Sara A. Mahmoud, Abeer Elkhoely, Elsayed K. El-Sayed, Amany A. E. Ahmed

**Affiliations:** Pharmacology and Toxicology Department, Faculty of Pharmacy, Helwan University, Ein Helwan, Cairo, Egypt

**Keywords:** Gastric ulcer, Ethanol, Pioglitazone, Ligustrazine, SIRT1

## Abstract

Gastric ulcer is a disturbing disease that impacts many people worldwide. Pioglitazone (Piog), a thiazolidinedione, and ligustrazine (Ligu), a natural component of *Ligusticum chuanxiong* possess gastroprotective properties. However, the underlying mechanism is not well elucidated. The present study aimed to investigate the gastroprotective effects of Piog (15 mg/kg, p.o.), Ligu (15 mg/kg, p.o.), and their combination against ethanol-induced gastric ulcer in rats. Omeprazole (10 mg/kg) was used as a standard. Pre-treatment for 7 days with Piog, Ligu, and (Piog+Ligu) effectively alleviated ethanol-predisposed oxidative stress and inflammation through restoring HO-1, GSH, and SOD tissue levels and decreasing elevated MDA, TNF-α, ICAM, I-NOS, and IL-1β contents. Moreover, Piog, Ligu, and (Piog+Ligu) markedly inhibited the ethanol-induced increase of gastric NF-KB and BAX. In contrast, this pre-treatment regimen significantly accelerated protein expression of SIRT1, Nrf2, and Bcl-2, along with autophagic proteins, ATG5 and Beclin. Interestingly, macroscopic, histopathological examination and mucin content were in harmony with previous results, where pre-treatment with Piog, Ligu, and (Piog+Ligu) showed a declined mucosal injury as evidenced by the remarkable decrease of the ulcer area percentage by 62.3%, 38.7%, and 91.2%, respectively, compared to the ethanol-ulcerated group. In conclusion, Piog and Ligu exhibited remarkable gastroprotective properties. Our study was the first to show that Piog, Ligu, and (Piog+Ligu) ameliorated oxidative stress, inflammation, and apoptosis and accelerated the autophagic process via the upregulation of the upstream SIRT1 protein. It is worth mentioning that future studies are needed to pave the way for the clinical use of Piog and Ligu as gastro-protective agents.

**Supplementary Information:**

The online version contains supplementary material available at 10.1007/s00210-024-03026-6.

## Introduction

Gastric ulcer is one of the most common chronic gastrointestinal disorders affecting about 5 to 10% of populations all over the world (Salari et al. 2021). The pathophysiology of gastric ulcer is attributed to the disrupted equilibrium between the endogenous destructive factors which include, gastric acid, pepsin, reactive oxygen species (ROS), and the defensive cytoprotective factors including prostaglandins (PGs), mucus-bicarbonate barrier, enzymatic and nonenzymatic antioxidants (AlRashdi et al. 2012). Other factors such as *Helicobacter pylori* infection, stress, smoking, non-steroid anti-inflammatory drugs (NSAIDs), and excessive alcohol intake also potentiate the risk of gastric ulcer (Desai et al. 1997; Guzmán-Gómez et al. 2018).

Acid-suppressive drugs including proton pump inhibitors (PPI) and H_2_ receptor blockers are the most widely pharmacological classes used for the treatment of gastric ulcers. However, these drugs exert many side effects such as impotence and gynecomastia which have been reported with cimitadine (H_2_-blocker) (Kuna et al. 2019). Nowadays, the H_2_-blocker ranitidine is not available in the market because of its possible carcinogenic effect (Nabil et al. 2021). On the other hand, the long-term use of PPIs is associated with kidney disease, fractures, vitamin B12, calcium, and magnesium deficiencies (Sivri 2004). Accordingly, a profound understanding of gastric ulcer pathogenesis and investigation of more effective gastroprotective agents is essential.

The pathophysiological effects of ethanol on gastric mucosa are inter-related. Ethanol affects the vascular endothelial cells of the gastric mucosa leading to hypoxia, associated with aggressive production of reactive oxygen species (ROS) (Suzuki et al. 2012). Previous studies reported decreased levels of anti-oxidant Nrf2, and HO-1 in ethanol-induced gastric injury experimental models (Raish et al. 2021, Shams and Eissa 2022). In the same context, downregulated Nrf2 aggravates inflammation through the enhancement of NF-κB and its down-stream inflammatory mediators (Yanaka 2018). Moreover, several studies have asserted the involvement of apoptosis in the pathophysiology of ethanol-induced gastric ulcer (Zhou et al. 2020, Shams and Eissa 2022). Apart from the previous pathophysiological aspects, autophagy is a restricted cellular degradation machinery that plays a mainstay role in conserving normal cellular physiology (Aman et al. 2021). Disregulated autophagy has been evidenced in ethanol-induced gastric injury models (Arab et al. 2022).

Sirtuins are family signaling proteins that are found in different species. In mammals, sirtuins are identified as seven isoforms (SIRT1-SIRT7) (Hegedűs et al. 2020). SIRT1 is NAD^+^-dependent histone deacetylase which is involved in many physiological and pathologic signaling pathways (Ugwu et al. 2017). Many studies reported that activation of SIRT1 can inhibit cell apoptosis and promote cell survival (Luo et al. 2001). Additionally, SIRT1 reduces cellular oxidative stress via modulating the bioavailability of NAD^+^ in tissues, thus providing a therapeutic prospect against metabolic and age-related diseases (Santos et al. 2016). In the same context, SIRT1 has been shown to ameliorate inflammation by interfering with NF-κB signaling pathway in pancreatic beta-cell destruction models in rats (Lee et al. 2009). Moreover, many studies have asserted the role of SIRT-1 activation in the promotion of the autophagic process in cardiomyocytes of hypoxic mice (Luo et al. 2019), and aging-related diseases (Wang et al. 2021). Taken together, SIRT1 may be an appealing protective and therapeutic target against gastric ulcers.

Pioglitazone (Piog) is a member of thiazolidinediones, insulin-sensitizing drugs, which improve insulin sensitivity and dyslipidemia in type 2 diabetics (Ding et al. 2005). Pioglitazone elicits its effect by activating the peroxisome proliferator–activator receptor (PPAR-γ) (Konturek et al. 2003). The expression of PPAR-γ is not restricted to the adipose tissue and pancreas but is also expressed in the liver, stomach, small intestine, and colon indicating its possible contribution to multiple biological processes (Braissant et al. 1996). A previous study showed that pioglitazone enhanced gastric ulcer healing in rats through the activation of PPAR-ɣ receptors and hence the acceleration of angiogenesis at ulcer margin (Brzozowski et al. 2005). In addition, the study performed by Konturek et al. (2003) was the first to approve the gastroprotective effect of pioglitazone in acetic acid-induced ulcers in rats. Pioglitazone demonstrated an anti-inflammatory effect via suppression of pro-inflammatory cytokines, cyclooxygenase-2, and inducible nitric oxide synthase (iNOS) (Konturek et al. 2003). Later on, the gastroprotective effect of pioglitazone was documented in cholestatic rats by decreasing IL-1β and nitric oxide levels (Moezi et al. 2014). Interestingly, pioglitazone exhibited neuroprotective effects in streptozotocin-induced diabetes in rats via modulation of the brain SIRT-1 signaling pathway (Tork et al. 2019).

Ligustrazine (Ligu), a bioactive constituent extracted from the dry rhizome of *Ligusticum wallichii*, has been traditionally used in China for the treatment of many diseases (Zhao et al. 2016). Ligustrazine showed gastroprotective effects against indomethacin-induced gastric ulcer in rats, partly through its antioxidant, anti-inflammatory and angiogenic effects (AlKreathy et al. 2020). Ligustrazine showed a nephroprotective effect against cisplatin-induced oxidative stress and apoptosis (Liu et al. 2008). Moreover, ligustrazine attenuated atherosclerotic lesions in rats and suppresses oxidative stress by enhancing total antioxidant capacity, superoxide dismutase 1 (SOD1) activity, and decreasing MDA level (Jiang et al. 2011). The anti-inflammatory activity of ligustrazine was documented via its ability to suppress the NF-κB pathway (Liu et al. 2017; Yu et al. 2018). Of interest, ligustrazine showed a therapeutic potential effect via the regulation of the SIRT1/NF-κB pathway in Freund’s complete adjuvant-induced arthritis model (Li et al. 2019).

It is worth-mentioning that both pioglitazone and ligustrazine have been shown to possess ameliorative properties against gastric injury‏ (Moezi et al. 2013; AlKreathy et al. 2020); however, the exact underlying mechanism is not well elucidated.

The current study aimed to assess the gastroprotective effect of pioglitazone, a synthetic drug and ligustrazine, a natural plant constituent against ethanol-induced gastric ulcer in rats. The underlying antioxidant, anti-inflammatory, and antiapoptotic mechanisms were explored. Besides, a special focus on the involvement of autophagy and SIRT1 signaling pathways in the pathogenesis of gastric injury induced by ethanol was inspected.

## Materials and methods

### Animals

Adult male *Sprague Dawley* rats (180–200 g) were acquired from the breeding unit of the Egyptian Organization of Biological Products and Vaccines (Helwan, Egypt). Rats were randomized and housed using twenty cages (3 rats/cage) under controlled environmental conditions at a constant temperature (25ºC ± 2ºC) and 12-h light/dark cycles. They were allowed free access to a standard pellet diet (Meladco Feed Company, Obour City, Cairo, Egypt) for a week acclimatization period before the experimental procedures and given tap water *ad libitum*. Animal care and experimental protocols were approved by the animal care and use committee at the Faculty of Pharmacy, Helwan University (Protocol #: 08A2021). All animal handling and procedures were performed according to the Guide for the Care and Use of Laboratory Animals published by the US National Institute of Health (NIH publication No. 85-23, revised 1996).

### Drugs

Ligustrazine, omeprazole, and absolute ethanol were purchased from Sigma Aldrich Chemical Co (St. Louis, MO, USA). Pioglitazone was purchased from Al-Arabia Chemical Co (Cairo, Egypt).

### Experimental design

**Fig. 1:**
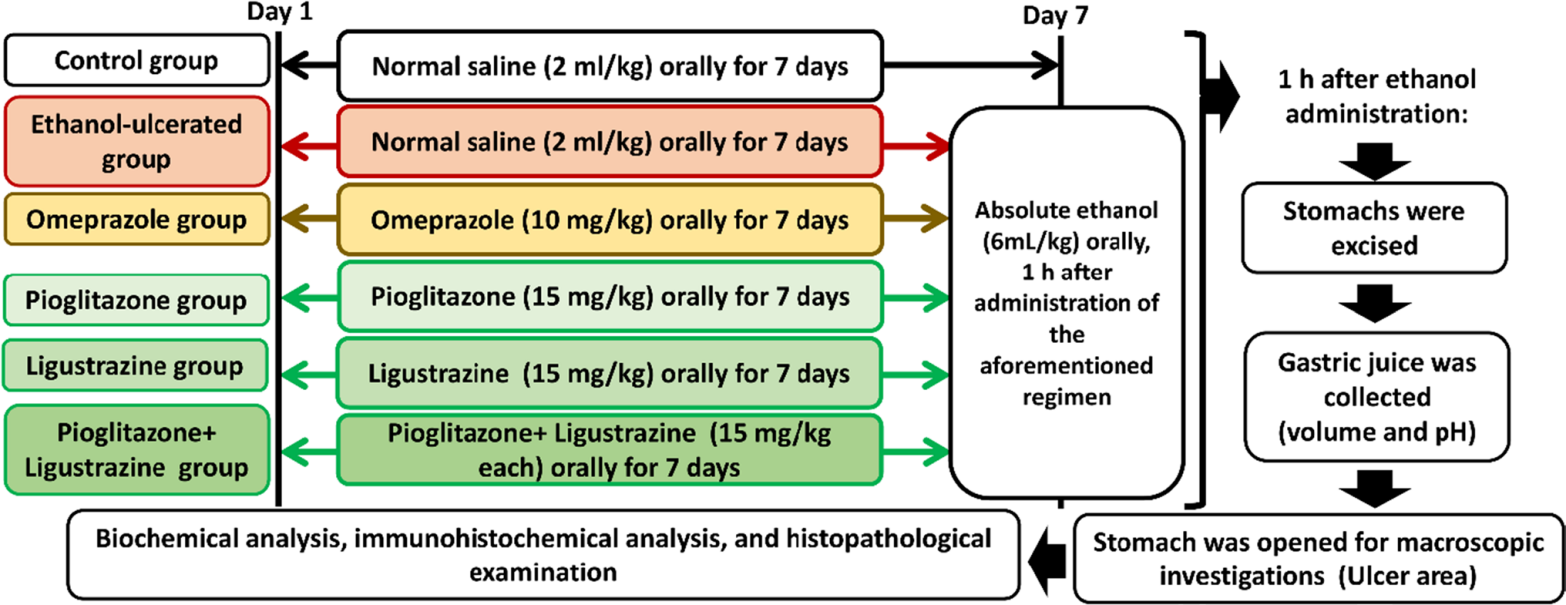
A schematic illustration of the experimental design

Sixty adult male rats were randomly divided into 6 groups (*n* = 10). Group I: Normal control group, Group II: Ethanol ulcerated group, Group III: Omeprazole (standard) + ethanol group, Group IV: Pioglitazone + ethanol group, Group V: Ligustrazine + ethanol group, Group VI: (Pioglitazone+ Ligustrazine) + ethanol group. Rats in groups I and II received normal saline (2 ml/kg) orally for 7 days using gastric gavage, while those in groups III, IV, V, and VI were administered orally with omeprazole (10 mg/kg) (Pandian et al. 2002), pioglitazone (15 mg/kg) (Moezi et al. 2014), ligustrazine (15 mg/kg) (AlKreathy et al. 2020), (pioglitazone + ligustrazine) (15 mg/kg each), respectively for 7 days. On the seventh day, absolute ethanol (6ml/kg) was administered to fasting rats 1 h after the administration of the aforementioned regimen except for the normal control group (Fig. 1).

### Tissue collection and preparation

One hour after ethanol administration, rats were sacrificed, and their stomachs were excised. Gastric contents were collected for the determination of gastric volume (ml) and pH. After that, the stomach was opened along the greater curvature, washed with normal saline, and photographed for macroscopic investigations (ulcer area). For each group, each stomach was divided into two halves. For histopathological and immunohistochemical examinations, six halves were fixed in 10 % neutral buffered formalin. Another six halves were homogenized in phosphate buffer (0.1M, pH 7.4) to obtain 10% w/v tissue homogenate, and then centrifugated at 4,000 rpm for 10 min. The cleared supernatant was preserved at - 80°C for the assessment of biochemical parameters. Finaly, from each group, six halves of stomach were homogenized in RIPA lysis buffer, and the clear supernatants were used western blot analysis.

### Measurement of gastric juices volume and pH

The collected gastric contents (mL) were measured and then centrifuged for 10 min at 1000 rpm and 4°C. The obtained supernatants were used for the determination of gastric pH using a digital pH Meter (6173pH, Jenco Electronics Co, Texas, USA).

### Morphological examination and evaluation of gastroprotection

Each stomach was opened along the greater curvature, washed with normal saline, and placed on a flat plate for examination of gross lesions. Photographs (10-megapixel 5 × zoom) were captured using a digital camera and areas of ulcerated lesions were calculated using Image analysis software (Image J, 1.46a). The ulcerated area percentages relative to the total area of the stomach were estimated.

### Biochemical analyses

#### Determination of gastric oxidative stress markers

Malondialdehyde (MDA) (nmol/mL) level and superoxide dismutase (SOD) (u/mL) in the stomach were assayed colorimetrically using diagnostic kits (MyBioSource, Cat# MBS841695-100, Cat# MBS480450 and BioVision, Cat# K335, USA respectively) according to the manufacturer’s protocols. Where, reduced glutathione (GSH) (μg/mL) and hemeoxygenase-1(HO-1) (ng/ml) levels in the stomach were measured using ELISA kits (MyBioSource, Cat# MBS724319 and BioVision, Cat# E4525, USA).

#### Determination of gastric inflammatory markers

The levels of tumor necrosis factor-alpha (TNF-α) (pg/mL), interleukin-1β (IL-1β) (pg/ml), intracellular adhesion molecule-1 (ICAM-1) (pg/mL), and inducible nitric oxide synthase (iNOS) (μg/mL) in the gastric tissue homogenates were assayed using ELISA kits (ThermoFisher,Cat.No.ERA56RBX5, Thermofisher, Cat# BMS630TEN, BioVision, Cat# K7163***,*** USA and MyBioSource, Cat# MBS023874, USA respectively).

#### Determination of gastric protein expression by western blotting

Western blotting was performed for nuclear factor erythroid 2-related factor 2 (Nrf2); an upstream regulator of anti-oxidant markers, phosphorylated nuclear factor-kappa B (p-NF-κB); an upstream regulator of inflammatory mediators, sirtuin 1 (SIRT1); an upstream regulator to the pathophysiological aspects under investigation, i.e. oxidative stress, inflammation, apoptosis and autophagy, autophagy-related 5 (ATG5), and Beclin-1; mainstay mediators of the autophagic process. Cytosolic and nuclear proteins of gastric tissue were extracted using a RIBA lysis buffer (150 mMNaCl, 1 .0% NP-40 or 0. 1% Triton X-100, 0.5% sodium deoxycholate, 0.1% SDS (sodium dodecyl sulphate) and 50 mM Tris-HCl, pH was adjusted at 8.0) and Bradford Protein Assay Kit (Bio Basic Inc.). Isolated proteins were electrophoretically separated on sodium dodecyl sulfate-polyacrylamide gel and then transferred to polyvinylidene difluoride membrane using Bio-Rad Trans-Blot Turbo instrument. After that, membrane was incubated with rabbit polyclonal antibodies β-actin, Nrf2 (ThermoFisher, Cat# PA5-68817, USA), p-NF-κB (ThermoFisher, Cat# PA5-16545, USA), SIRT1 (ThermoFisher, Cat# PA5, USA), ATG5 (ThermoFisher, Cat# OSA00021W) and Beclin-1 (ThermoFisher, Cat# OSB00001G, USA) (Antibodies’ dilution 1:2000) in TBS-T(Tris-buffered saline with Tween 20). Incubation was done overnight in each primary antibody solution, against the blotted target protein, at 4°C. After washing, a horseradish peroxidase-conjugated secondary anti-rabbit antibody (1: 10 000 dilution) (Novus Biologicals, Littleton, CO, USA) was added and incubated for 2 h then rinsed three times. The chemiluminescent substrate (Clarity TM Western ECL substrate - BIO-RAD, Cat# 170-5060, USA) was applied to the blot according to the manufacturer’s recommendation. Visualization of the protein bands was done using a CCD camera-based imager. Image analysis software was used to read the band intensity of the target proteins against the control sample after normalization by β-actin on the Chemi Doc MP imager.

### Histopathological examinations

Stomach tissue samples were fixed in 10% neutral buffered formalin for 72 h. Samples were then dehydrated by immersion in serial concentrations of alcohol (70–100%) and cleared in xylene. Afterward, samples were embedded in paraffin wax blocks. Sections 4 μm thick were stained with hematoxylin and eosin (H&E) as a standard method for morphological examination, and stained by Alcian blue pH 2.5 for demonstration of acidic mucins (Culling 2013). Finally, samples were examined by Full HD microscopic imaging system (Leica Microsystems GmbH, Germany). (Scale bar: 200 µm (×100) and 50 µm (×400)).

### Immunohistochemical examinations

For immunohistochemical analysis, 5 μm thick paraffin-embedded gastric sections were prepared. Tissue sections were treated with 0.3% H_2_O_2_ for 20 min, followed by incubation with anti-BAX primary antibody (Thermo Fisher, Catalog # MA5 -14003, USA) (1:100) and anti-Bcl2 (Thermo Fisher, Catalog # PA1-30411, USA) (1:100) for overnight at 4°C. Afterward, sections were washed out with PBS and incubated with a secondary antibody HRP Envision kit (DAKO) for 20 min. Tissue sections were then rinsed gently in PBS and incubated with diaminobenzidine (DAB) substrate chromagen for 15 min following washing and counterstaining with hematoxylin. The sections were then dehydrated, cleared in xylene, and covered and slipped for microscopic examination.

For quantification, the area percentage of immunohistochemical expression levels of mucosal BAX and Bcl2 proteins were calculated using a Full HD microscopic imaging system operated by the Leica application module for histological analysis (Leica Microsystems GmbH, Germany) (Scale bar: 50 µm, ×400).

### Statistical analysis

The data were expressed as mean value ± SEM. Statistical analysis and graphical representations were performed using GraphPad Prism, version 8 (GraphPad Software Inc., San Diego, California, USA), using a one-way analysis of variance (ANOVA), followed by a Tukey’s test to determine the statistical significance between various groups. The value *p* < 0.05 was considered as significant.

## Results

### Effect of pioglitazone and ligustrazine and their combination on gastric juice volume and pH

Oral administration of absolute ethanol significantly (*p* < 0.05) increased the gastric juice volume by 4 folds, while significantly (*p* < 0.05) decreased the pH of the gastric juice by 49.8%, compared with the control group. However, pre-treatment with omeprazole, pioglitazone (Piog) and ligustrazine (Ligu) and their combination (Piog+Ligu) significantly (*p* < 0.05) decreased the volume by 27.8%, 40.5%, 15.2%, and 39.1% respectively; and significantly (*p* < 0.05) increased the pH of gastric juice by 1.5 folds, 1.8 folds, 1.7 folds, and 2 folds, respectively, compared to the ethanol-ulcerated untreated group (Table 1). Interestingly, the drug combination showed a significant (*p* < 0.05) decrease in gastric juice volume by 15.7% and an increase in its pH by 1.4 folds as compared to the omeprazole standard group.

**Table 1:** Effect of pioglitazone (Piog) and ligustrazine (Ligu) and their combination (Piog+Ligu) on gastric juice volume, pH, and the percentage of ulcerated area in ethanol-induced gastric ulcer in rats

Groups	Gastric juice volume (mL)	Gastric juice acidity (pH)	% Ulcerated area
Control	1.31±0.07	3.78±0.009	0.00±0.00
Ethanol	5.28±0.07^a,c,d^	1.89±0.06^a,c,d^	28.99±2.23^a,c,d^
Omeprazole	3.81±0.09^a,b,d^	2.79±0.08^a,b,d^	9.49±0.79^a,b,d^
Piog	3.41±0.21^a,b^	3.44±0.09^a,b,c,d^	10.94±0.70^a,b,d^
Ligu	4.47±0.12^a,b,c,d^	3.22±0.06^a,b,c,d^	17.77±1.17^a,b,c,d^
Piog + Ligu	3.21±0.11^a,b,c^	3.80±0.01^b.c^	2.54±0.23^b.c^

Data presented as Mean ± SE, *n* = 6. Statistical comparisons among groups were carried out using ANOVA test followed by Tukey-Kramer multiple comparison tests. a: significant from control, b: significant from ethanol, c: significant from omeprazole, d: significant from combination (Piog+Ligu)

### Effect of pioglitazone and ligustrazine and their combination on macroscopic examinations of gastric mucosa

**Fig. 2:**
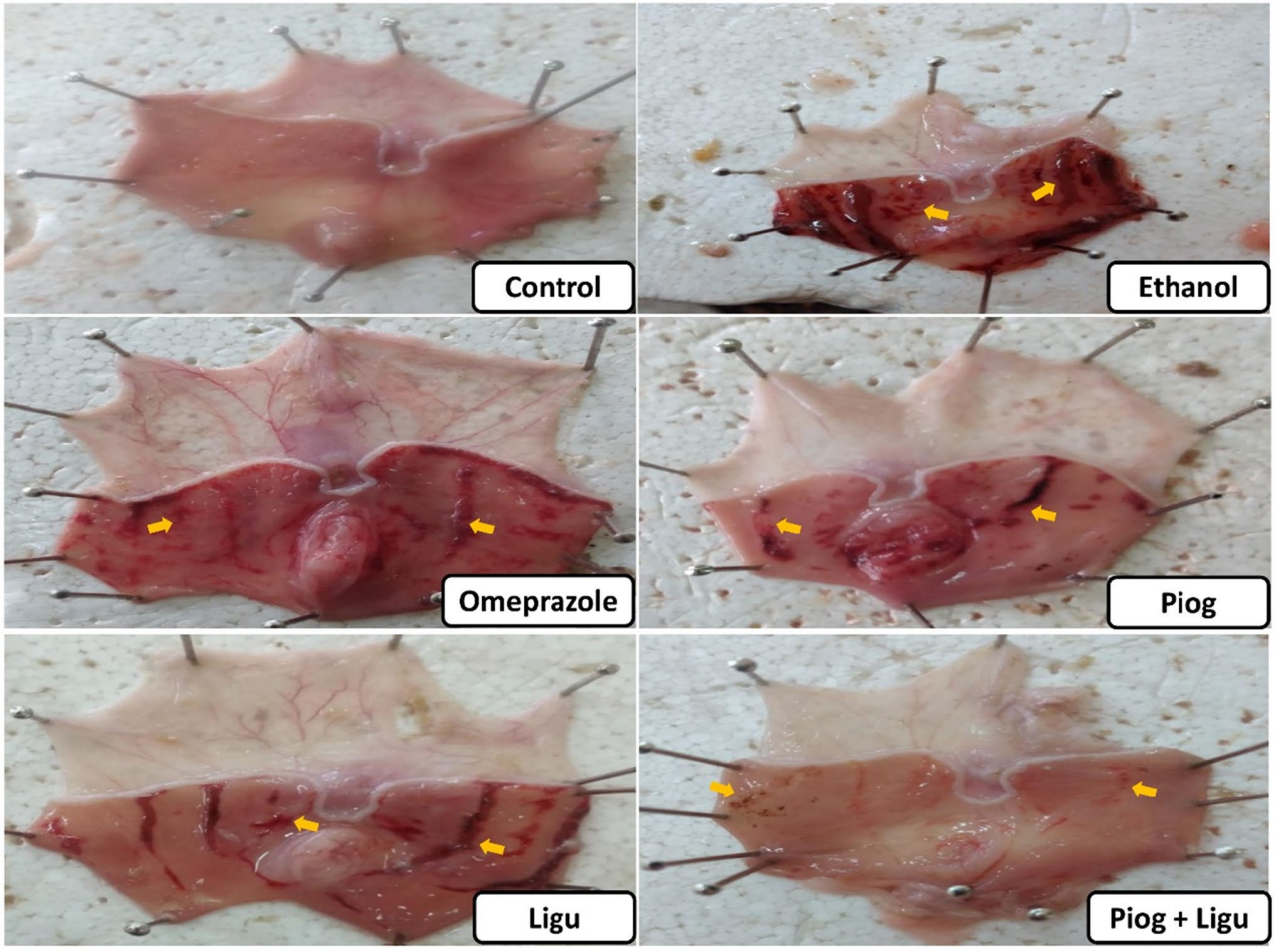
Photographic representation showed the effect of pioglitazone (Piog) and ligustrazine (Ligu) and their combination (Piog+Ligu) on gross gastric lesions in ethanol-induced gastric ulcer in rats. Yellow arrow: ulcerated areas. (Scale= 1 mm)

The ethanol-ulcerated group showed a significant (*p* < 0.05) increase in the percentage of ulcer area compared to the control group (Table 1). In contrast, pre-treatment with omeprazole, pioglitazone (Piog) and ligustrazine (Ligu) and their combination (Piog+Ligu) showed a less mucosal injury as evidenced by a significant (*p* < 0.05) decrease in the ulcer area percentage by 667.3%, 62.3%, 38.7%, and 91.2%, respectively, compared to the ethanol-ulcerated untreated group. Moreover, the combination of (Piog+Ligu) showed a better protective effect indicated by a significant (*p* < 0.05) decreased ulcer area by 73.2% as compared to the omeprazole standard group (Fig. 2).

### Effect of pioglitazone and ligustrazine and their combination on gastric oxidative stress parameters and antioxidant enzymes

Treatment with ethanol induced oxidative stress in the gastric tissue as evidenced by the significant increase in MDA level and decrease in GSH, HO-1 contents and SOD activity.

**Fig. 3:**
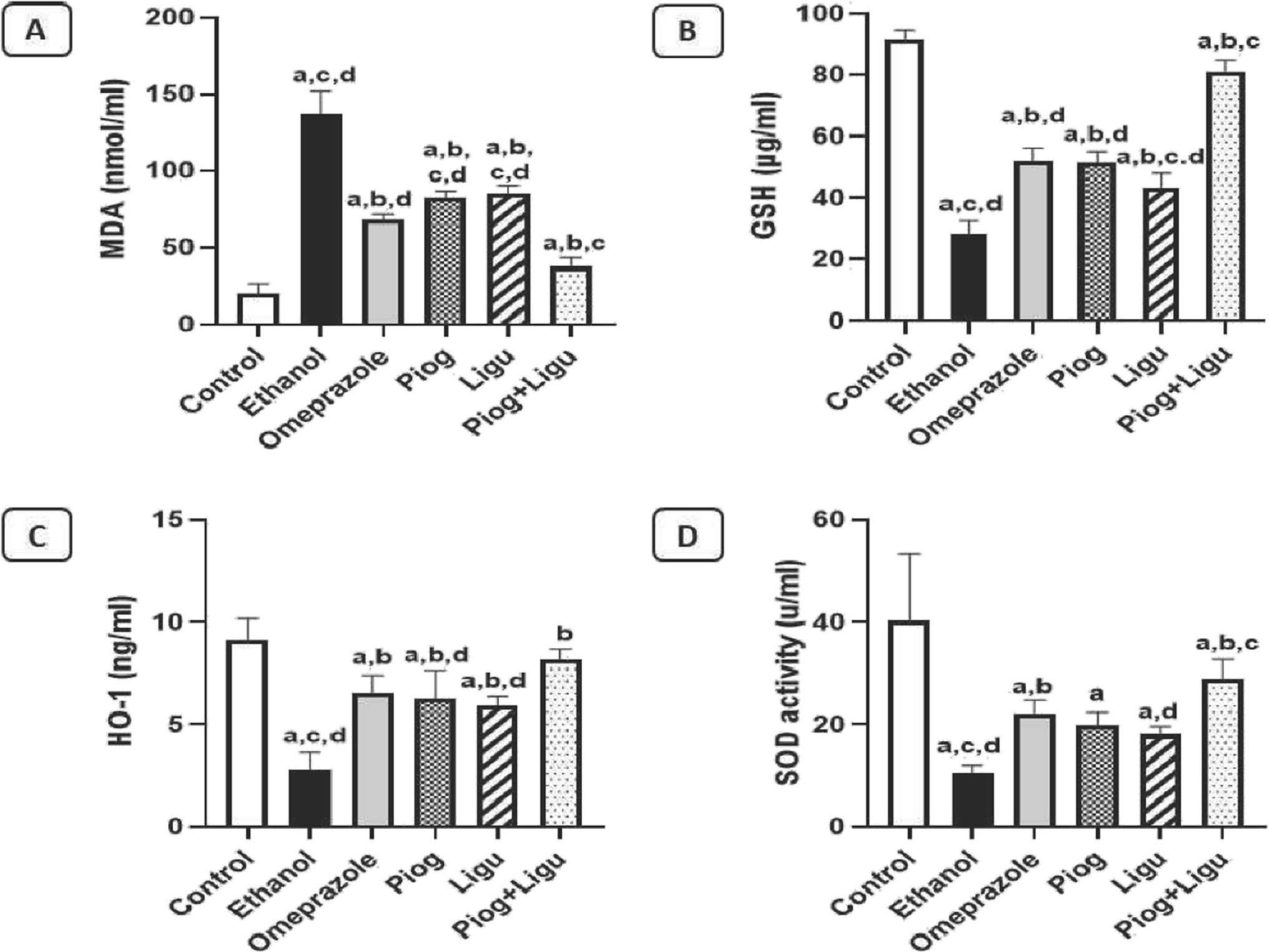
Effect of pioglitazone (Piog) and ligustrazine (Ligu) and their combination (Piog+Ligu) on gastric oxidative stress parameters in ethanol-induced gastric ulcer in rats. (**A**) MDA: malondialdehyde, (**B**) GSH: reduced glutathione, (**C**) HO-1: hemeoxygenase-1and (**D**) SOD: superoxide dismutase. Data presented as Mean ± SE, *n* = 6. Statistical comparisons among groups were carried out using ANOVA test followed by Tukey-Kramer multiple comparison tests. a: significant from control, b: significant from ethanol, c: significant from omeprazole, d: significant from combination (Piog+Ligu)

MDA is the main reactive metabolite produced by ROS-induced lipid peroxidation, which can inactivate many cellular proteins by forming protein cross-linkages. The ethanol-ulcerated group showed a significant (*p* < 0.05) increase in MDA level, indicating lipid peroxidation and oxidative stress. Pre-treatment with omeprazole, pioglitazone (Piog) and ligustrazine (Ligu) and their combination (Piog+Ligu) showed a significant (*p* < 0.05) decrease in MDA level by 50.4%, 39.8%, 37.6%, and 73%, respectively, compared to the ethanol-ulcerated untreated group (Fig. 3A). Interestingly, the combination (Piog+Ligu) significantly (*p* < 0.05) decreased MDA level as compared to the omeprazole group by 44.6%.

GSH is a non-enzymatic antioxidant molecule that fights cellular oxidative stress through scavenging free radicals and reactive species. The ethanol-ulcerated group exhibited a significant (*p* < 0.05) decrease in GSH content by 68.9% compared to the control group. Meanwhile, pre-treatment with omeprazole, pioglitazone (Piog) and ligustrazine (Ligu) and their combination (Piog+Ligu) significantly (*p* < 0.05) increased the GSH content by 1.8 folds, 1.8 folds, 1.5 folds, and 2.8 folds, respectively, compared to the ethanol-ulcerated untreated group (Fig. 3B). Moreover, the combination (Piog+Ligu) significantly (*p* < 0.05) increased GSH level by 1.6 folds compared to the omeprazole group.

In the same context, HO-1 and SOD; enzymatic anti-oxidant enzymes as enzymatic antioxidant enzymes were also investigated. Our results showed a significant decrease in HO-1 content and SOD activity in the ethanol-ulcerated untreated group by 69.9% and 73.8%, respectively, compared to the control group. However, pre-treatment with omeprazole, pioglitazone (Piog) and ligustrazine (Ligu) and their combination (Piog+Ligu) exerted a significant (*p* < 0.05) increase in HO-1 level by 2.4, 2.3, 2.2, and 3 folds, respectively; and in SOD activity by 2.1, 1.9, 1.7, and 2.7 folds, respectively, compared to the ethanol-ulcerated untreated group (Fig. 3C and D).

### Effect of pioglitazone and ligustrazine and their combination on gastric inflammatory markers

**Fig. 4:**
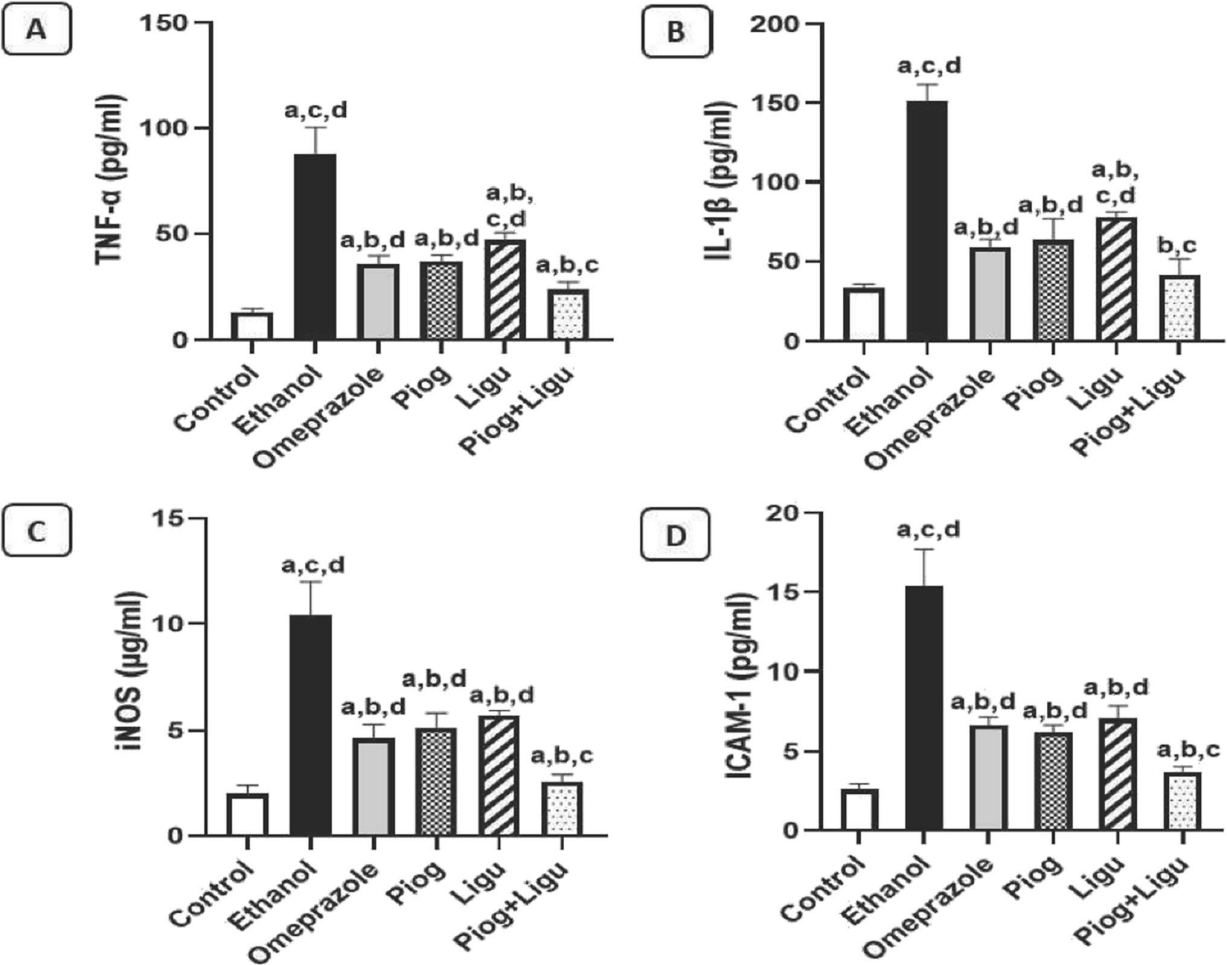
Effect of pioglitazone (Piog) and ligustrazine (Ligu) and their combination (Piog+Ligu) on gastric mucosal inflammatory markers in ehanol-induced gastric-ulcer in rats. (**A**) TNF-α**:** Tumor necrosis factor α, (**B**) IL-1β**:** interleukin-1β, (**C**) iNOS: inducible nitric oxide synthase and (**D**) ICAM-1**:** intracellular adhesion molecule-1. Data presented as Mean ± SE, *n* = 6. Statistical comparisons among groups were carried out using ANOVA test followed by Tukey-Kramer multiple comparison tests. a: significant from control, b: significant from ethanol, c: significant from omeprazole, d: significant from combination (Piog+Ligu)

In the current work, levels of pro-inflammatory mediators, TNF-α, IL-1β, ICAM-1 and iNOS were significantly (*p* < 0.05) elevated in ethanol-ulcerated untreated group by 6.9 folds, 4.6 folds, 5.9 folds and 5.2 folds respectively, compared to control rats. Oral administration of omeprazole, pioglitazone (Piog) and ligustrazine (Ligu) and their combination (Piog+Ligu) significantly (*p* < 0.05) decreased the levels of TNF-α by 59.8%, 58.1%, 46.7%, and 73.3% respectively; and IL-1β by 60.9%, 58%, 48.5%, and 72.6%, respectively, compared to the ethanol-ulcerated untreated group (Fig. 4A and B). Of interest, rats which received combined drugs (Piog+Ligu) showed a significant (*p* < 0.05) decrease in TNF-α and IL-1β by 33.7% and 29.9%, respectively, compared to the (standard drug) omeprazole group. Meanwhile, oral administration of omeprazole, pioglitazone (Piog) and ligustrazine (Ligu) and their combination (Piog+Ligu) significantly (*p* < 0.05) decreased the levels of ICAM-1 by 56.7%, 59.5%, 54.2%, and 76% respectively; and iNOS by 55. %, 50.5%, 45%, and 75.3% respectively, compared to the ethanol-ulcerated untreated group (Fig. 4C and D). It is worth-noting that rats received (Piog+Ligu) showed a significant (*p* < 0.05) decrease in ICAM-1 and iNOS by 44.5% and 44.6%, respectively, compared to the standard omeprazole group.

### Effect of pioglitazone and ligustrazine on the expression of p-NF-κB, Nrf2, SIRT1, ATG5, and Beclin-1 evaluated by western blotting

**Fig. 5:**
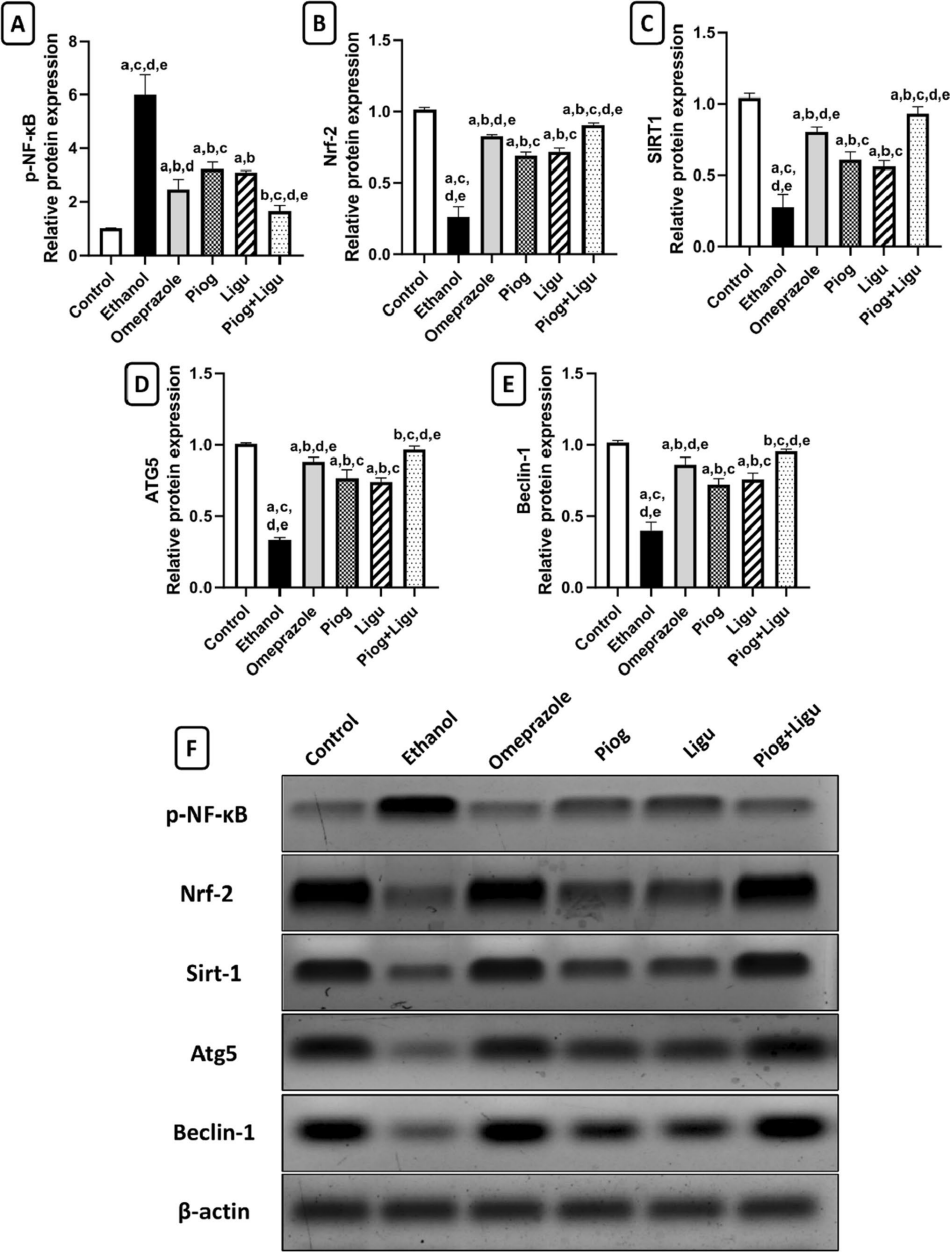
Effect of pioglitazone (Piog) and ligustrazine (Ligu) and their combination (Piog+Ligu) on the expression of p-NF-κB); an upstream regulator of inflammatory mediators, Nrf2; an upstream regulator of anti-oxidant markers, SIRT1; an upsrtream regulator to the pathophysiological aspects under investigation, i.e., oxidative stress, inflammation, apoptosis and autophagy, ATG5), and Beclin-1; mainstay mediators of the autophagic process in ethanol-induced gastric ulcer in rats. (**A**) Nrf2: nuclear factor erythroid 2-related factor 2, (**B**) p-NF-κB: phosphorylated nuclear factor-kappa B, (**C**) SIRT1: sirtuin 1, (**D**) ATG5: autophagy-related 5, (**E**) Beclin-1 and (**F**) Western blot autoradiography for the five parameters and beta-actin. Data presented as Mean ± SE, *n* = 6 Statistical comparisons among groups were carried out using ANOVA test followed by Tukey-Kramer multiple comparison tests. a: significant from control, b: significant from ethanol, c: significant from omeprazole, d: significant from combination (Piog+Ligu)

In the present study, the ethanol-ulcerated untreated group significantly (*p* < 0.05) increased the expression of p-NF-κB; an upstream inflammatory mediator, by 5.9 folds while significantly (*p* < 0.05) decreased the expression of Nrf2 by 74.6 %, respectively, compared to the control group. In contrast, pre-administration of omeprazole, pioglitazone, ligustrazine, and combination significantly (*p* < 0.05) decreased the expression of p-NF-κB by 58.9%, 46.2%, 48.3%, and 72.5%, respectively, and significantly increased (*p* < 0.05) the expression of Nrf2; an upstream anti-oxidant regulator, by 3.2 folds, 2.7 folds, 2.8 folds, and 3.5 folds, respectively, compared to the ethanol-ulcerated untreated group (Fig. 5A, B and F).

Moreover, the ethanol-ulcerated untreated group significantly (*p* < 0.05) decreased the expression of SIRT1 an upsrtream regulator to the pathophysiological aspects under investigation, i.e., oxidative stress, inflammation, apoptosis and autophagy, by 73.8% compared to the control group. On the other hand, pre-administration of omeprazole pioglitazone (Piog) and ligustrazine (Ligu) and their combination (Piog+Ligu) significantly (*p* < 0.05) increased the expression of SIRT1 by 2.9 folds, 2.2 folds, 2 folds, and 3.4 folds, respectively, compared to the ethanol-ulcerated untreated group (Fig. 5C and F).

Finally, the expression of ATG5 and Beclin-1; mainstay mediators of the autophagic process was significantly decreased in ethanol-ulcerated untreated group by 66.4% and 60.3%, respectively, compared to the control group. Meanwhile, pre-administration of omeprazole, pioglitazone (Piog) and ligustrazine (Ligu) and their combination (Piog+Ligu) significantly (*p* < 0.05) increased the expression of ATG5 by 2.6 folds, 2.3 folds, 2.2 folds, and 2.9 folds, respectively; and Beclin-1 by 2.1 folds, 1.8 folds, 1.9 folds, and 2.4 folds, respectively, compared to the ethanol-ulcerated untreated group (Fig. 5D, E, F).

Notably, the combination group (Piog+Ligu) showed a significant (*p* < 0.05) decrease p-NF-κB expression by 33.1% and a significant (*p* < 0.05) increase in Nrf2, SIRT1, ATG5, and Beclin-1 protein expressions by 1.09 folds, 1.2 folds, 1.09 folds, and 1.1 folds, respectively, compared to the standard omeprazole group.

### Effect of pioglitazone and ligustrazine and their combination on the immunohistochemical expression of BAX and Bcl2 of the gastric mucosa

**Fig. 6:**
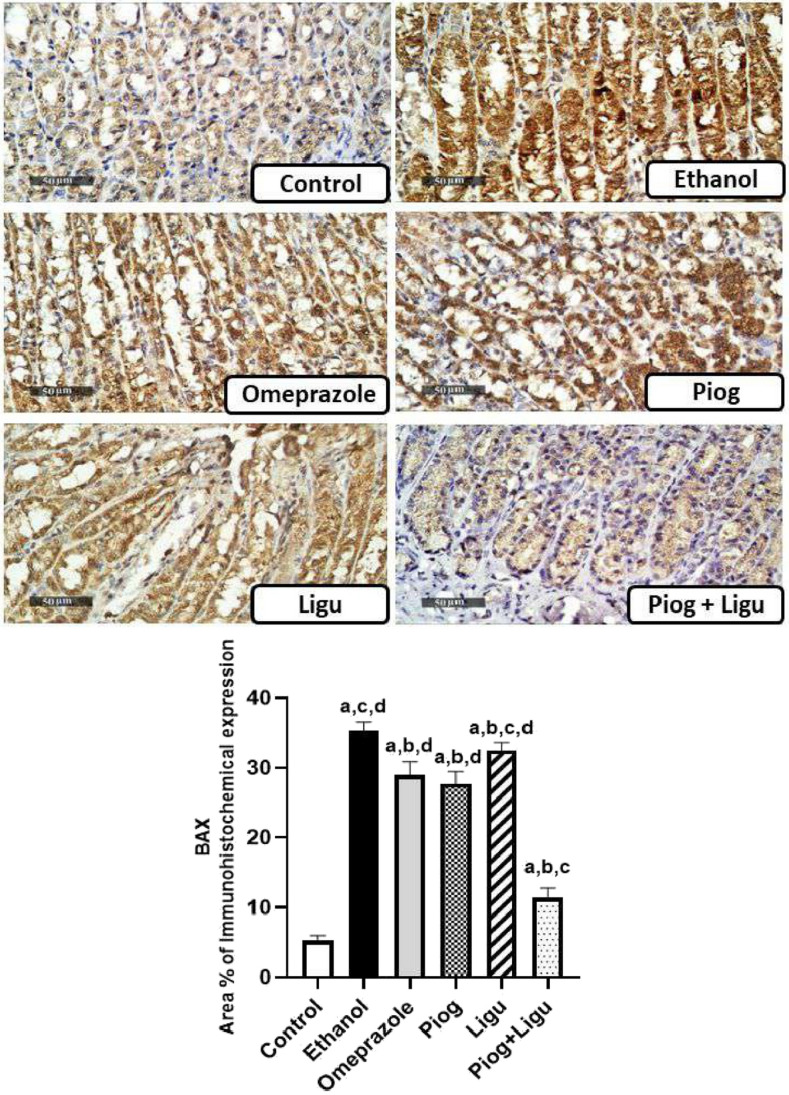
Effect of pioglitazone (Piog) and ligustrazine (Ligu) and their combination (Piog+Ligu) on the immunohistochemical expression of BAX; a pro-apoptotic marker, in the gastric mucosa in ethanol-induced gastric ulcer in rats. (Scale bar: 50 µm, ×400). Data presented as Mean ± SE, *n* = 6. Statistical comparisons among groups were carried out using ANOVA test followed by Tukey-Kramer multiple comparison tests. a: significant from control, b: significant from ethanol, c: significant from omeprazole, d: significant from combination (Piog+Ligu)

**Fig. 7:**
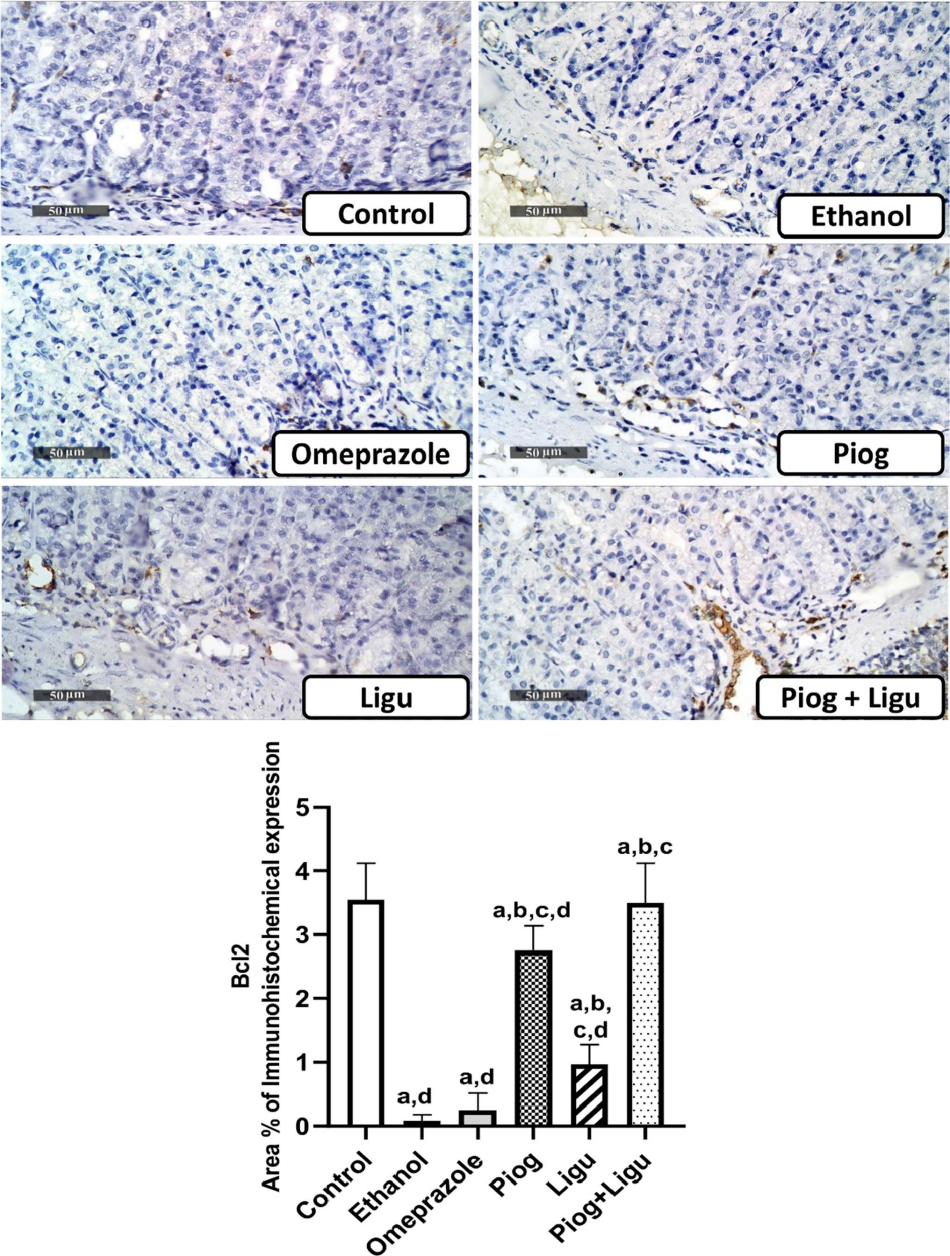
Effect of pioglitazone (Piog) and ligustrazine (Ligu) and their combination (Piog+Ligu) on the immunohistochemical expression of Bcl-2an anti-apoptotic marker, in the gastric mucosa in ethanol-induced gastric ulcer in rats. (Scale bar: 50 µm, ×400). Data presented as Mean ± SE, *n* = 6. Statistical comparisons among groups were carried out using ANOVA test followed by Tukey-Kramer multiple comparison tests. a: significant from control, b: significant from ethanol, c: significant from omeprazole, d: significant from combination (Piog+Ligu)

As shown in Figs. 6 and 7, oral administration of absolute ethanol significantly (*p* < 0.05) increased the expression of BAX; a pro-apoptotic marker by 6.8 folds and significantly (*p* < 0.05) decreased Bcl2; an anti-apoptotic marker, expression by 97.7 % compared to the control group. On the other hand, pretreatment with omeprazole, pioglitazone (Piog) and ligustrazine (Ligu) and their combination (Piog+Ligu) significantly (*p* < 0.05) declined BAX expression by 17.9%, 21.6%, 7.9%, and 67.8%, respectively and significantly (*p* < 0.05) enhanced the expression of Bcl2 by 3 folds, 33 folds, 11.6 folds, and 42 folds, respectively, compared to the ethanol-ulcerated untreated group. To be noted, the combination (Piog+Ligu) showed a significant (p < 0.05) increase in Bcl2 by 14 folds and a significant (*p* < 0.05) decrease in BAX by 60.7% compared to the (standard) omeprazole group.

### Effect of pioglitazone and ligustrazine and their combination on mucin content of the gastric mucosa

**Fig. 8:**
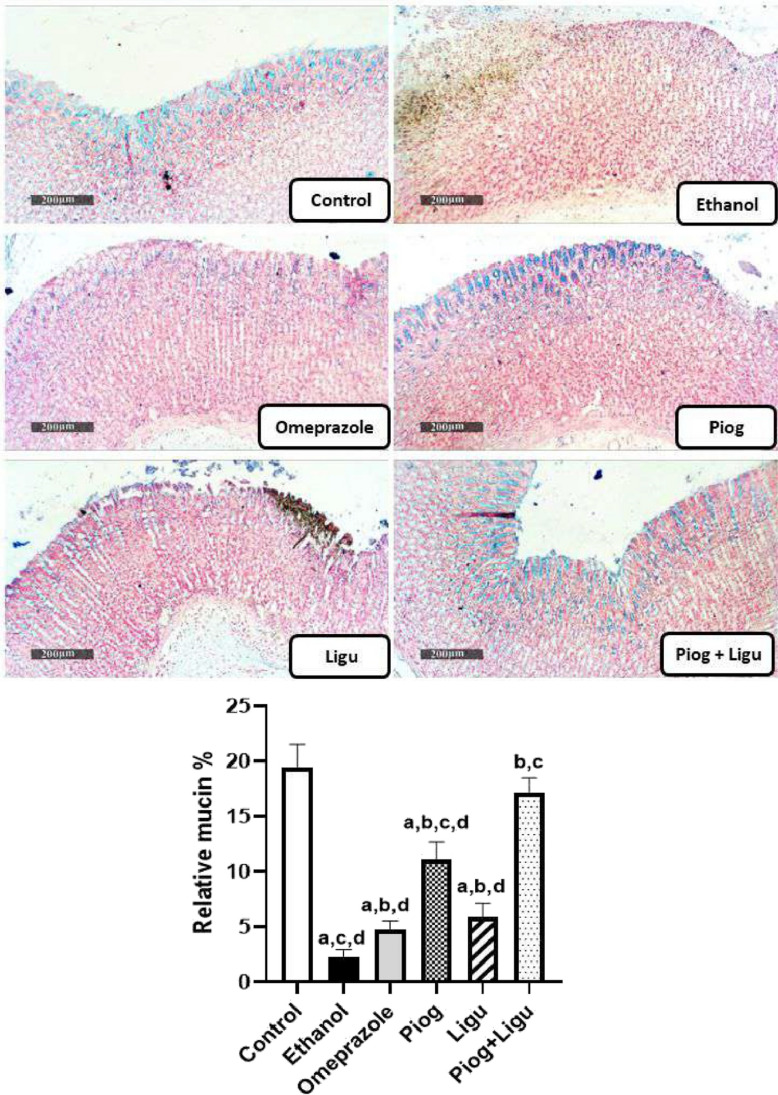
Effect of pioglitazone (Piog) and ligustrazine (Ligu) and their combination (Piog+Ligu) on mucin content of the gastric mucosa in ethanol-induced gastric ulcers in rats (Scale bar: 50 µm, x100). Data presented as Mean ± SE, *n* = 6. Statistical comparisons among groups were carried out using ANOVA test followed by Tukey-Kramer multiple comparison tests. a: significant from control, b: significant from ethanol, c: significant from omeprazole, d: significant from combination (Piog+Ligu)

As illustrated in Fig. 8, the ethanol-ulcerated untreated group showed a significant decrease in the amount of acidic mucin by 88.2% compared to the control group. In contrast, a significant increase in acidic mucin level was observed in groups treated with omeprazole, pioglitazone (Piog) and ligustrazine (Ligu) and their combination (Piog+Ligu) by 2.1 folds, 4.9 folds, 2.6 folds, and 7.6 folds, respectively, compared to the ethanol-ulcerated untreated group. Notably, the amount of mucin in the gastric mucosa of the combination group (Piog+Ligu) was significantly increased by 3.6 folds compared to the (standard) omeprazole group.

### Effect of pioglitazone and ligustrazine and their combination on histopathological examination of the gastric mucosa

**Fig. 9:**
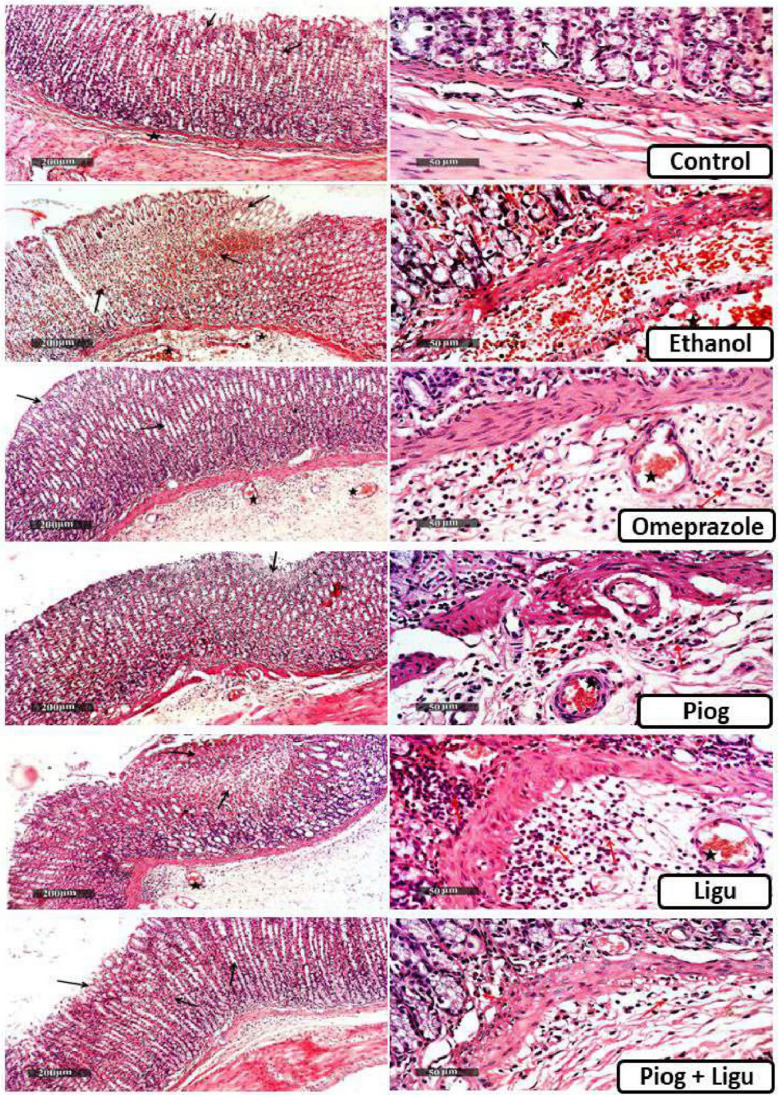
Effect of pioglitazone (Piog) and ligustrazine (Ligu) and their combination (Piog+Ligu) on histological features of stomach layers in ethanol-induced gastric ulcer in rats. Black arrow: multiple focal areas of mucosal hemorrhagic ulcerations with necrotic glandular tissue depress, red arrow: submucosal oedema with inflammatory cells infiltrates, star: congested blood vessels. (Scale bar: 200 µm (x100) and 50 µm (×400))

Regarding the ethanol-ulcerated untreated group, histopathological examinations of the gastric tissue samples showed multiple focal areas of mucosal hemorrhagic ulcerations with necrotic glandular tissue *(black arrow)*, submucosal edema with inflammatory cells infiltrates *(red arrow),* as well as moderate congested and dilated mucosal/ submucosal blood vessels *(star)* (Fig. 9) (Table 2). Conversely, the control sample showed normal morphological features of the gastric wall with well-organized intact glandular elements, epithelial tissues *(arrow),* submucosa with minimal inflammatory cell infiltrates *(star),* and vasculatures with the outer muscular coat. Pre-treatment with, omeprazole, pioglitazone and Ligustrazine ameliorated the structural changes, where samples showed moderate focal mucosal erosions *(black arrow),* moderate reduction of submucosal edema, and inflammatory cell infiltrates *(red arrow)* and some congested blood vessels *(star)*. Interestingly, combined drug (Piog+Ligu) samples showed almost intact gastric walls with normal organized morphological features including lining mucosa including glandular structures *(black arrow),* an almost intact submucosal layer with mild focal inflammatory cells infiltrates *(red arrow)* as well as mild edema.

**Table 2:** Effect of pioglitazone (Piog) and ligustrazine (Ligu) and their combination (Piog+Ligu) on lesion score records obtained from the histological examination in ethanol-induced gastric ulcer in rats

Groups	Control	Ethanol	Omeprazole	Piog	Ligu	Piog + Ligu
Erosion/ulceration	-	+++	-	+	++	-
Hemorrhagic patches	-	+++	-	-	-	-
Inflammatory cells infiltrate	-	++	+	+	++	+
Blood vessels congestion	-	++	+	+	++	+
Oedema	-	+++	++	++	++	+

Nil (-) = no lesion was evidenced, + = mild lesion records evidenced in less than 15% of examined tissue sections, ++ = moderate lesion records evidenced in 16–35% of examined tissue sections, +++ = severe lesion records evidenced in more than 35% of examined tissue sections. Piog: pioglitazone, Ligu: ligustrazine

## Discussion

The aim of the current study was to investigate the possible gastroprotective effects of pioglitazone and ligustrazine and their combination against ethanol-induced gastric ulcers in rats, as well as the underlying gastroprotective mechanisms were investigated with a special focus on the possible involvement of SIRT1 in the pathogenesis of ethanol-induced gastric injury.

Macroscopic inspection and histopathological examination have confirmed the gastric injury conferred by absolute ethanol administration. These results are in accordance with previous studies (Guler et al. 2022; Raish et al. 2021; Zhou et al. 2020). In contrast, pioglitazone and ligustrazine, alone and in combination remarkably ameliorated ethanol-induced ulceration as evidenced by retracted hemorrhagic lesions, edema and inflammation.

The involvement of oxidative stress, inflammation, apoptosis, along autophagy in the pathogenesis of gastric ulcer is undeniable (Suzuki et al. 2012; Watanabe et al. 2002; Szabó and Tarnawski 2000; Lu et al. 2021).

In our study, ethanol administration notably disrupted oxidant/antioxidant balance in favor of oxidation as revealed by a remarkable decrease of GSH, HO-1 tissue contents and SOD activity, along with a remarkable increase in MDA levels. In the same context, ethanol intake has aggravated the inflammatory response as evidenced by elevated tissue content of TNF-α, ICAM, INOS and IL-1β.

Previous studies have asserted the role of oxidative stress and inflammation in ethanol-induced gastric ulcer (Guzmán-Gómez et al. 2018; El-Naga 2015; Al Asmari et al. 2015). Moreover, ethanol administration resulted in elevated apoptosis as manifested by a decrease of antiapoptotic Bcl-2protein and a remarkable increase of proapoptotic Bax protein. In addition, ethanol predisposed notable decrease in the expression of Beclin-1 and ATG 5 which are essential autophagy proteins (Graf et al. 2009).

In agreement with our results, Zhou et al. (2020) showed that ethanol-induced gastric injury resulted in a significant rise in BAX and Caspase 3 protein expression, along with a notable decrease in protein expression of antiapoptotic Bcl-2.

Regarding autophagy which is considered a key player in the maintenance of cellular survival (Khandia et al. 2019), a previous study revealed that suppressed autophagy exacerbated gastric ethanol-induced oxidative damage (Chang et al. 2017).

On the other hand, pioglitazone, ligustrazine and their combination were able to significantly reverse the aforementioned parameters. Interestingly, the combination of both drugs superiorly restored oxidative stress, inflammation, apoptosis and autophagy markers to normal levels.

In an attempt to elucidate the underlying regulators via which both pioglitazone and ligustrazine have conferred protection, SIRT1 protein was investigated. SIRT1 protein confers protection through regulation of the gene expression of many proteins involved in the pathogenesis of many diseases and toxic injuries (Elibol and Kilic 2018; Ren et al. 2019).

Focusing on oxidative stress as a crucial pathogenic pathway, SIRT1 can upregulate the expression of NrF2, widely regarded as an inducer of antioxidant proteins such as SOD, GSH and HO-1 (Huang et al. 2015). In contrast, SIRT1 downregulates NF-κB protein expression which is a well-known master enhancer of inflammatory response (Rothgiesser et al. 2010; Hah et al. 2014). Taking a glimpse at autophagy, SIRT1 positively enhances the autophagic process through the deacetylation of many proteins, among which are Beclin-1 and autophagic-related genes such as ATG5 (Sacitharan et al. 2020; Ou et al. 2014).

According to the results of the present study, each of pioglitazone and ligustrazine, separately and concomitantly significantly enhanced the expression of SIRT1, NrF2, Beclin-1 and ATG5, whilst the expression of NF-κB was markedly reduced. Previous studies revealed that ligustrazine directly activates SIRT1 (Li et al. 2019; Lin et al. 2022). Moreover, pioglitazone was shown to upregulate SIRT1 through its agonist effect on PPAR-ɣ (Wang et al. 2018; Carvalho et al. 2021).

In addition, it was reported that the anti-inflammatory effect of pioglitazone against gastric ulcer was through decreasing IL-1β, TNF-α and NO levels. Moreover, the gastroprotective effect of pioglitazone was attributed to the role of PPAR-gamma as an angiogenesis enhancer (Brzozowski et al. 2005). Furthermore, Pioglitazone demonstrated a gastroprotective effect against ethanol induced ulcer via inhibition of nitric oxide synthase activity and decreasing the level of IL-1β and TNF-α (Moezi et al. 2013; Moezi et al. 2014).

**Fig. 10:**
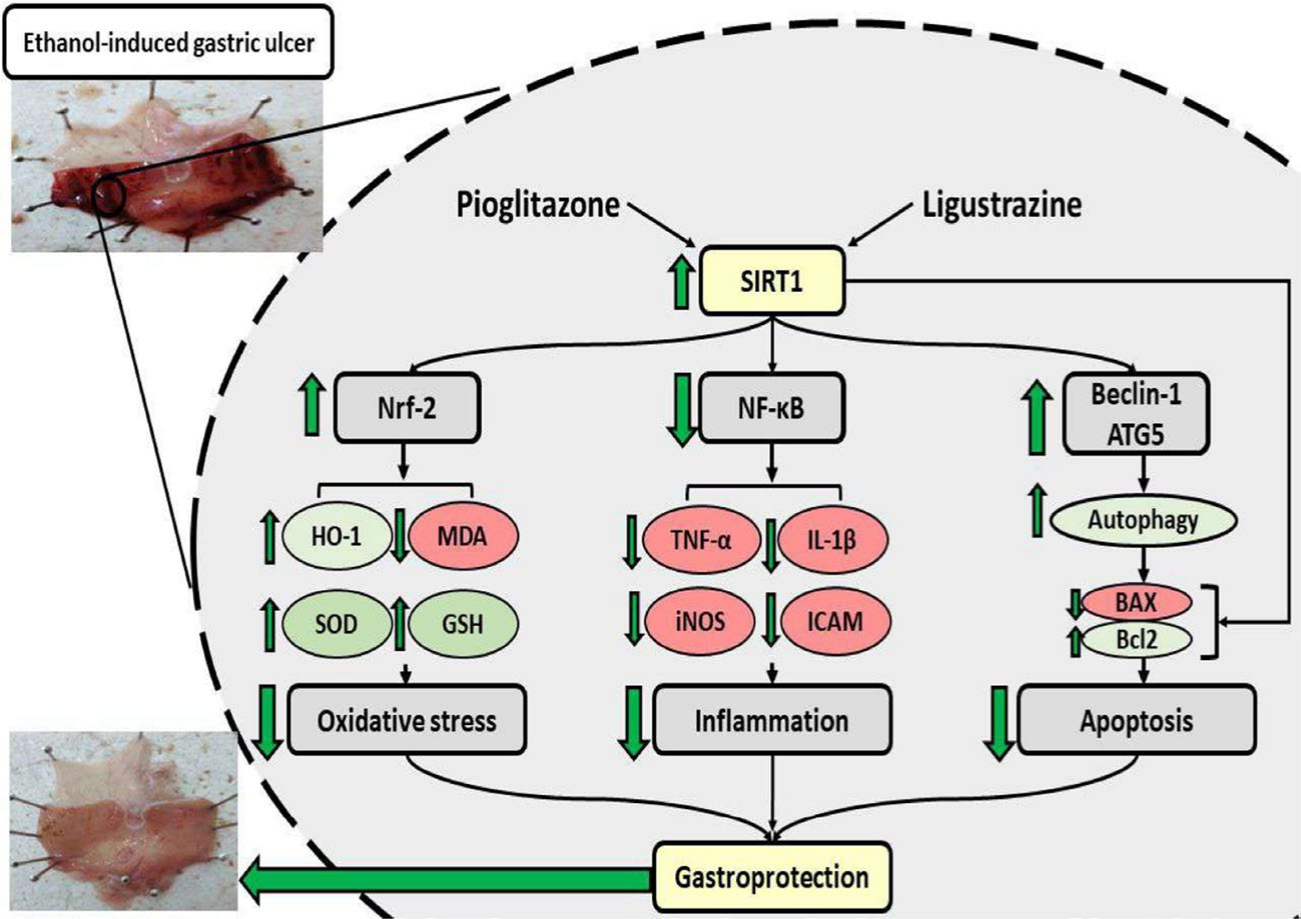
Summary of the gastroprotective effect of pioglitazone (Piog) and ligustrazine (Ligu) and their combination (Piog+Ligu) against ethanol-induced gastric ulcer in rats. Ethanol resulted in 1. Downregulated Nrf2 expression, attenuated HO-1, SOD, GSH contents and elevated MDA level. 2. Elevated NF-KB expression and its downstream inflammatory mediators; TNF-α, iNOS, ICAM-1 and IL-1β. 3. Inhibited expression of autophagic Beclin-1 and ATG5 proteins. 4. Enhanced pro-apoptotic Bax expression and inhibited expression of anti-apoptotic Bcl-2. Pioglitazone and ligustrazine conferred their gastroprotective effects partly via the augmented expression of SIRT1 protein. As a consequence, Pioglitazone and ligustrazine attenuated the previously-mentioned injurious manifestations, oxidative stress, inflammation and apoptosis. Moreover, Pioglitazone and ligustrazine improved autophagy*. Abbreviations:* (Nrf2), nuclear factor erythroid 2-related factor 2; (HO-1), heme oxygenase-1; (SOD), superoxide dismutase; (GSH), reduced glutathione; (MDA), malondialdehyde; (NF-κB (p65)), nuclear factor kappa-B (p65); (TNF-α), tumor necrosis factor alpha; (iNOS), inducible nitric oxide synthase; (ICAM-1), intracellular adhesion molecule-1; (IL-1β), interleukin-1β; INF-γ, (Bax), Bcl-2-associated X protein; (Bcl-2), B cell lymphoma 2; (ATG5), autophagy related 5; (SIRT1), Sirtuin 1

In conclusion, our results showed that pioglitazone, ligustrazine and their combination protected against ethanol-induced gastric ulcer. The protection was conveyed through upregulation of SIRT1 protein which in turn alleviated oxidative stress, inflammation, apoptosis and enhanced autophagy. It is worth noting that further mechanistic studies are needed to acknowledge the mainstay role of SIRT1 in conferring protective effects against gastric injury. Taken together, further studies, including clinical studies regarding dose and duration of treatment are needed to be investigated, along with the exploration of the gastro-protective effects of pioglitazone and ligustrazine in clinical settings in order to to set up a firm foundation for its feasible use clinically. Figure 10 elucidates the gastro-protective effects of pioglitazone and Ligustrazine against ethanol-induced gastric injury. In our study, pioglitazone and ligustrazine enhanced the expression of SIRT1 and accordingly ameliorated oxidative stress markers; MDA, GSH, SOD and HO-1, inflammatory mediators; TNF-α, IL-1β, iNOS and ICAM-1 and apoptotic markers caspase 3. In the same context, SIRT1 accelerated the autophagic process as evidenced by elevated ATG5 and Beclin-1 levels.

### Supplementary Information

Below is the link to the electronic supplementary material.

Supplementary file1 (JPG 997 KB)

Supplementary file2 (JPG 824 KB)

Supplementary file3 (JPG 984 KB)

Supplementary file4 (JPG 690 KB)

Supplementary file5 (JPG 1049 KB)

Supplementary file6 (JPG 1018 KB)

Supplementary file7 (JPG 1146 KB)

Supplementary file8 (JPG 1023 KB)

Supplementary file9 (JPG 1058 KB)

Supplementary file10 (JPG 1298 KB)

Supplementary file11 (JPG 1319 KB)

Supplementary file12 (JPG 1177 KB)

Supplementary file13 (JPG 1366 KB)

Supplementary file14 (JPG 1275 KB)

Supplementary file15 (JPG 787 KB)

Supplementary file16 (JPG 903 KB)

Supplementary file17 (JPG 886 KB)

Supplementary file18 (JPG 866 KB)

## Data Availability

Authors declare that they don’t have any research data outside the submitted manuscript file.

## Abbreviations

ATG5: Autophagy-related 5
BAX: Bcl-2-associated X protein
Bcl-2: B cell lymphoma 2
GSH: Reduced glutathione
HO-1: Hemeoxygenase-1
ICAM: Intracellular adhesion molecule-1
IL-1β: Interleukin-1beta
i-NOS: Inducible nitric oxide synthase
Ligu: Ligustrazine
MDA: Malondialdehyde
Nrf2: Nuclear factor erythroid 2-related factor 2
NSAIDs: Non-steroidal anti-inflammatory drugs
PGs: Prostaglandins
Piog: Pioglitazone
p-NF-κB: Phosphorylated nuclear factor-kappa B
PPIs: Proton pump inhibitors
ROS: Reactive oxygen species
SIRT1: Sirtuin 1
SOD: Superoxide dismutase
TNF-α: Tumor necrosis factor-alpha

## Declarations

### Limitations of the study

In clinical practice, Pioglitazone and ligustrazine may be taken as a combination therapy in case of type 2 diabetes mellites in which acute gastric inflammation and ulcer disease occur with high prevalence (Amr et al. 2010), a fact which dictates the study of the protective effect these two drugs against type2 diabetes mellitus-induced acute gastric ulcer.
