# Supplementary figures and images for: Enhanced upregulation of SIRT1 via pioglitazone and ligustrazine confers protection against ethanol-induced gastric ulcer in rats
Source: Naunyn Schmiedebergs Arch Pharmacol. 2024 Mar 5;397(8):6177–95. doi: 10.1007/s00210-024-03026-6 (PMC11329587; doi:10.1007/s00210-024-03026-6)

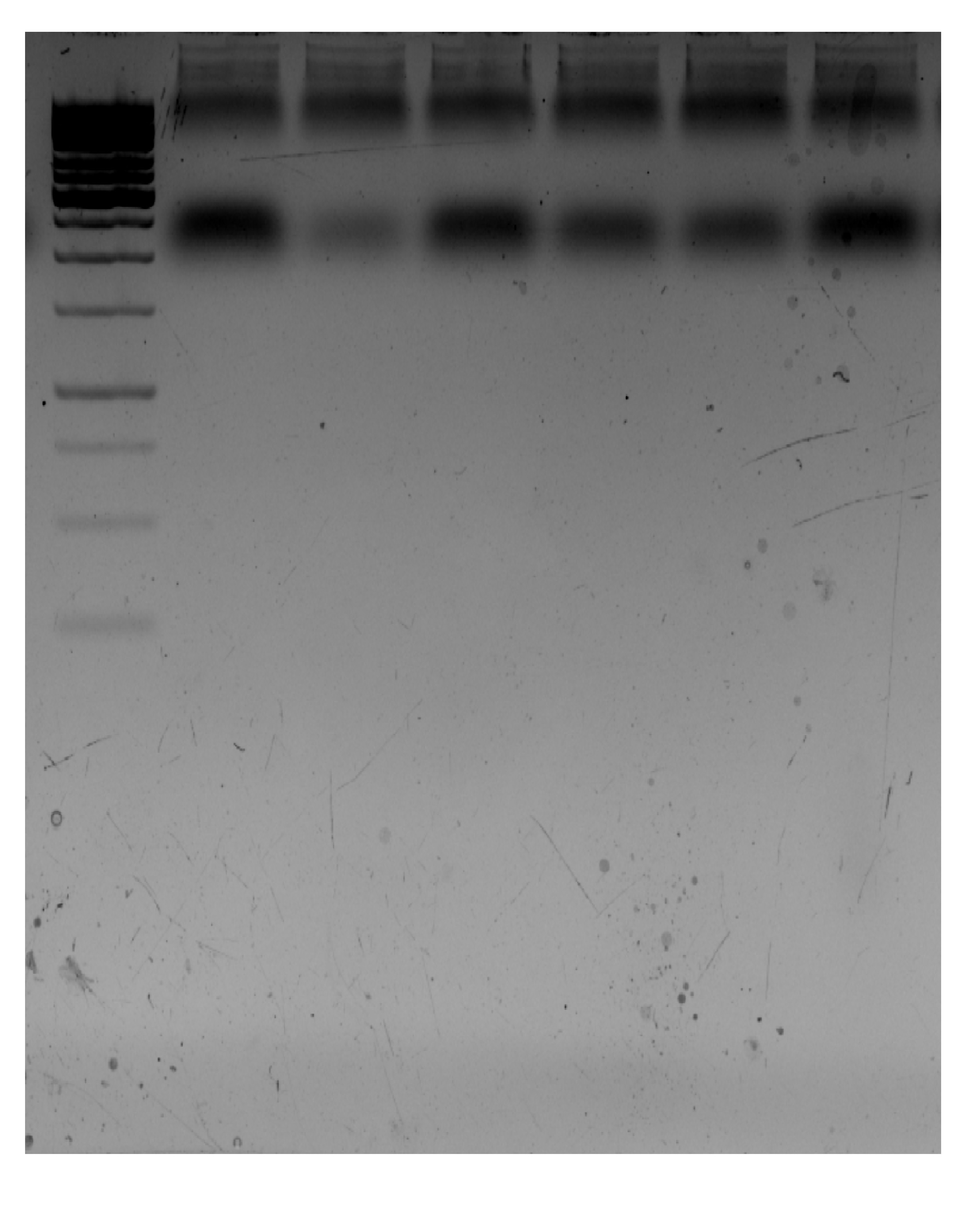

Supplement: Supplementary file 1 — Supplementary file1 (JPG 997 KB) [file 210_2024_3026_MOESM1_ESM.jpg]

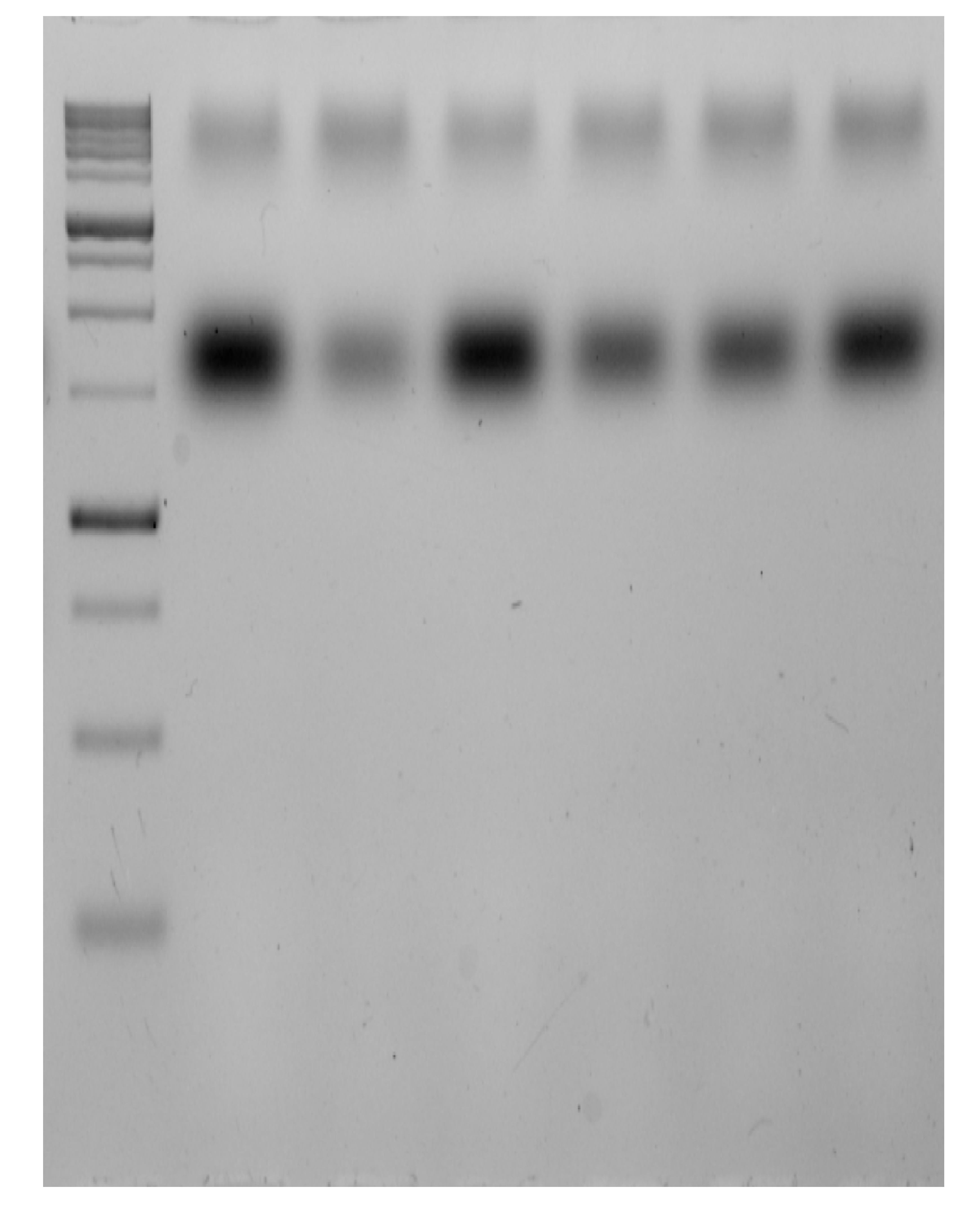

Supplement: Supplementary file 2 — Supplementary file2 (JPG 824 KB) [file 210_2024_3026_MOESM2_ESM.jpg]

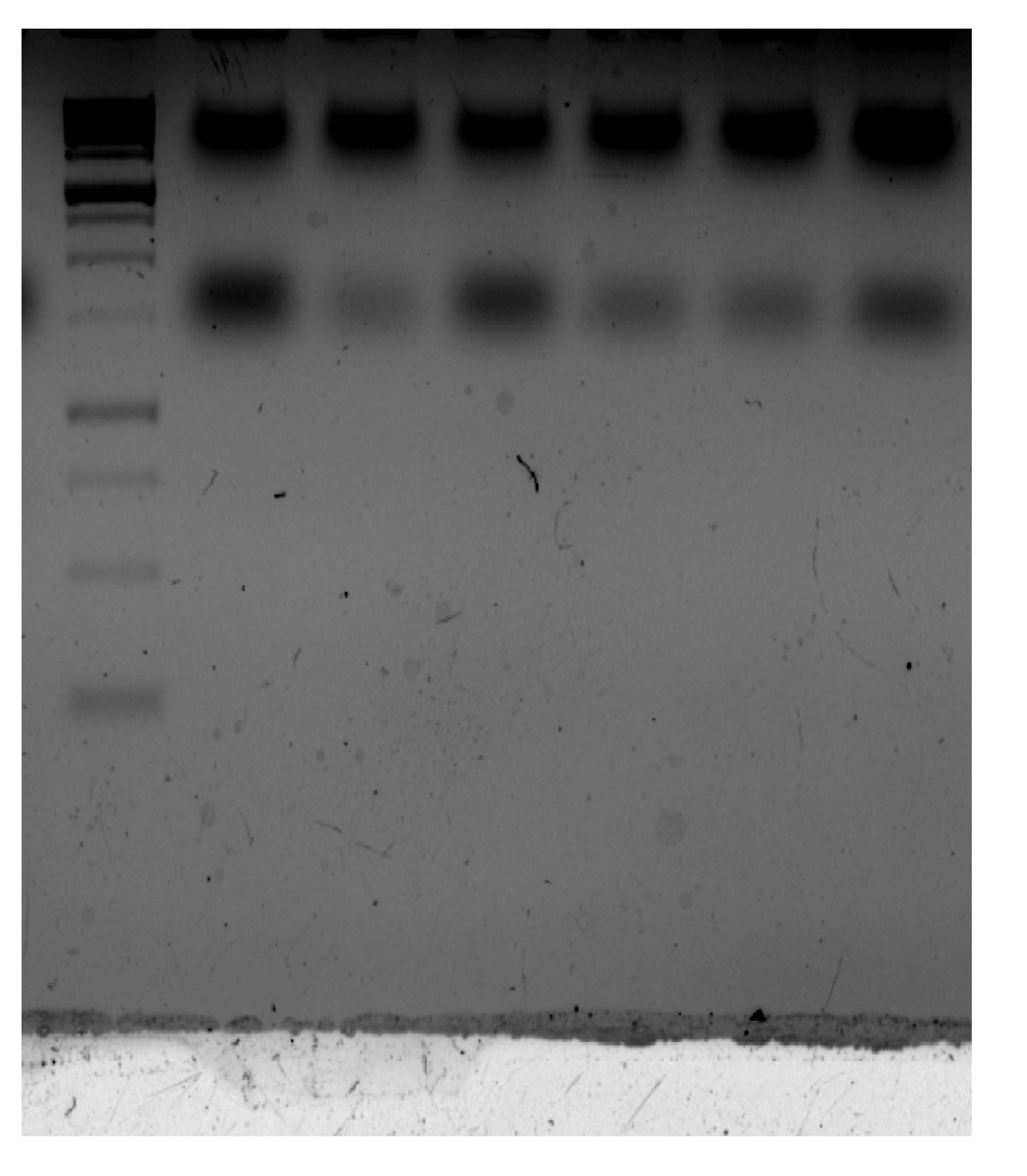

Supplement: Supplementary file 3 — Supplementary file3 (JPG 984 KB) [file 210_2024_3026_MOESM3_ESM.jpg]

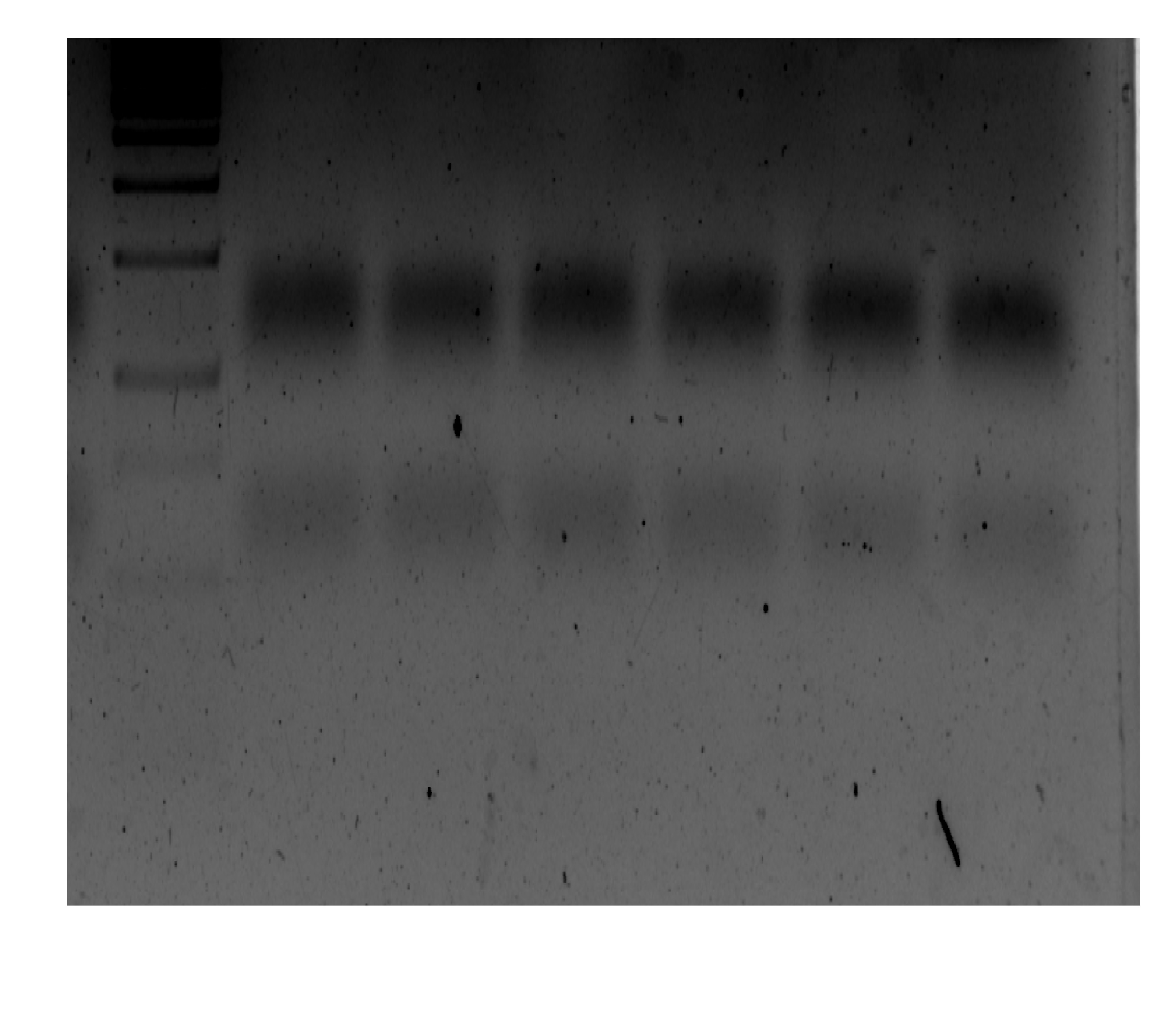

Supplement: Supplementary file 4 — Supplementary file4 (JPG 690 KB) [file 210_2024_3026_MOESM4_ESM.jpg]

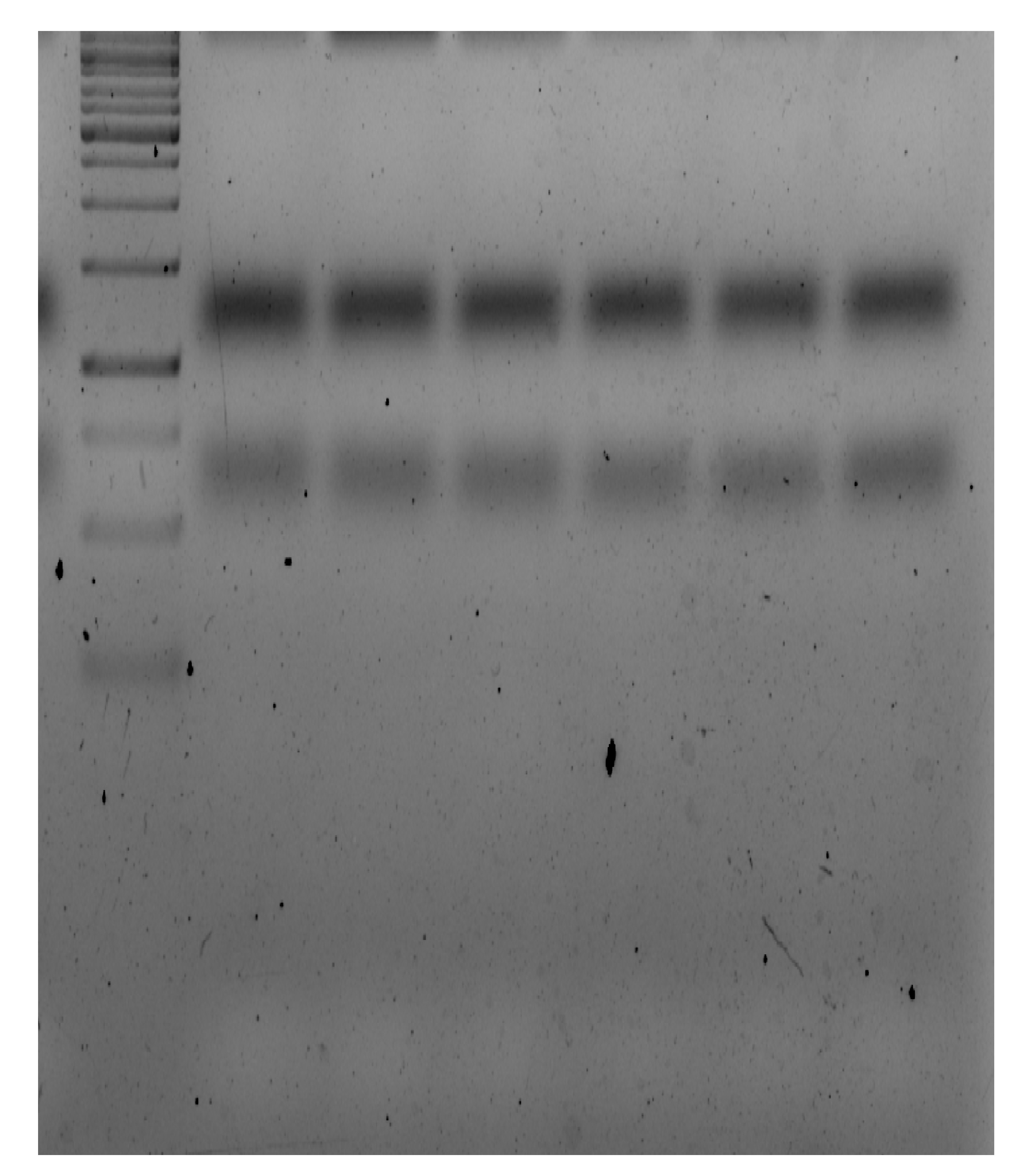

Supplement: Supplementary file 5 — Supplementary file5 (JPG 1049 KB) [file 210_2024_3026_MOESM5_ESM.jpg]

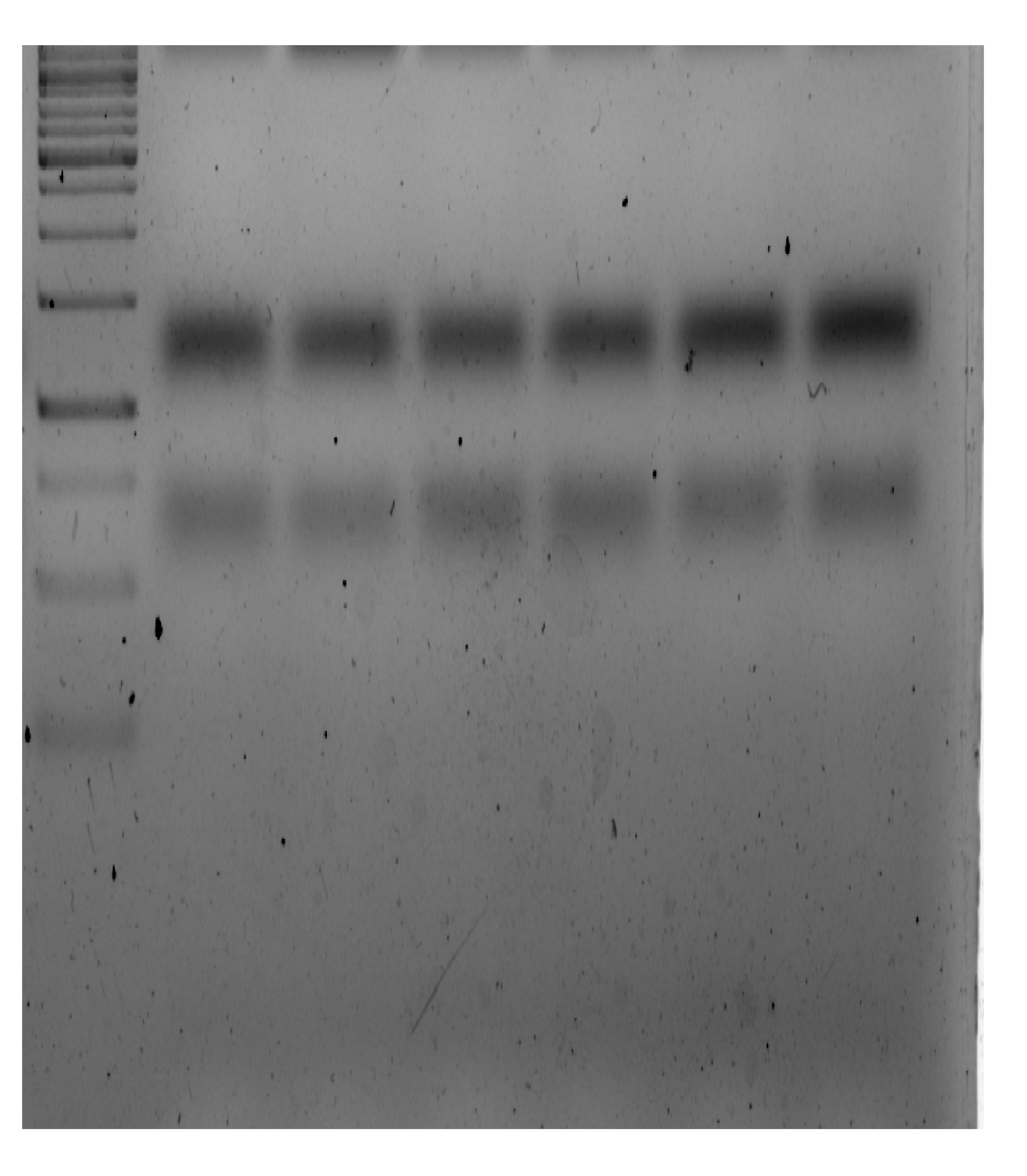

Supplement: Supplementary file 6 — Supplementary file6 (JPG 1018 KB) [file 210_2024_3026_MOESM6_ESM.jpg]

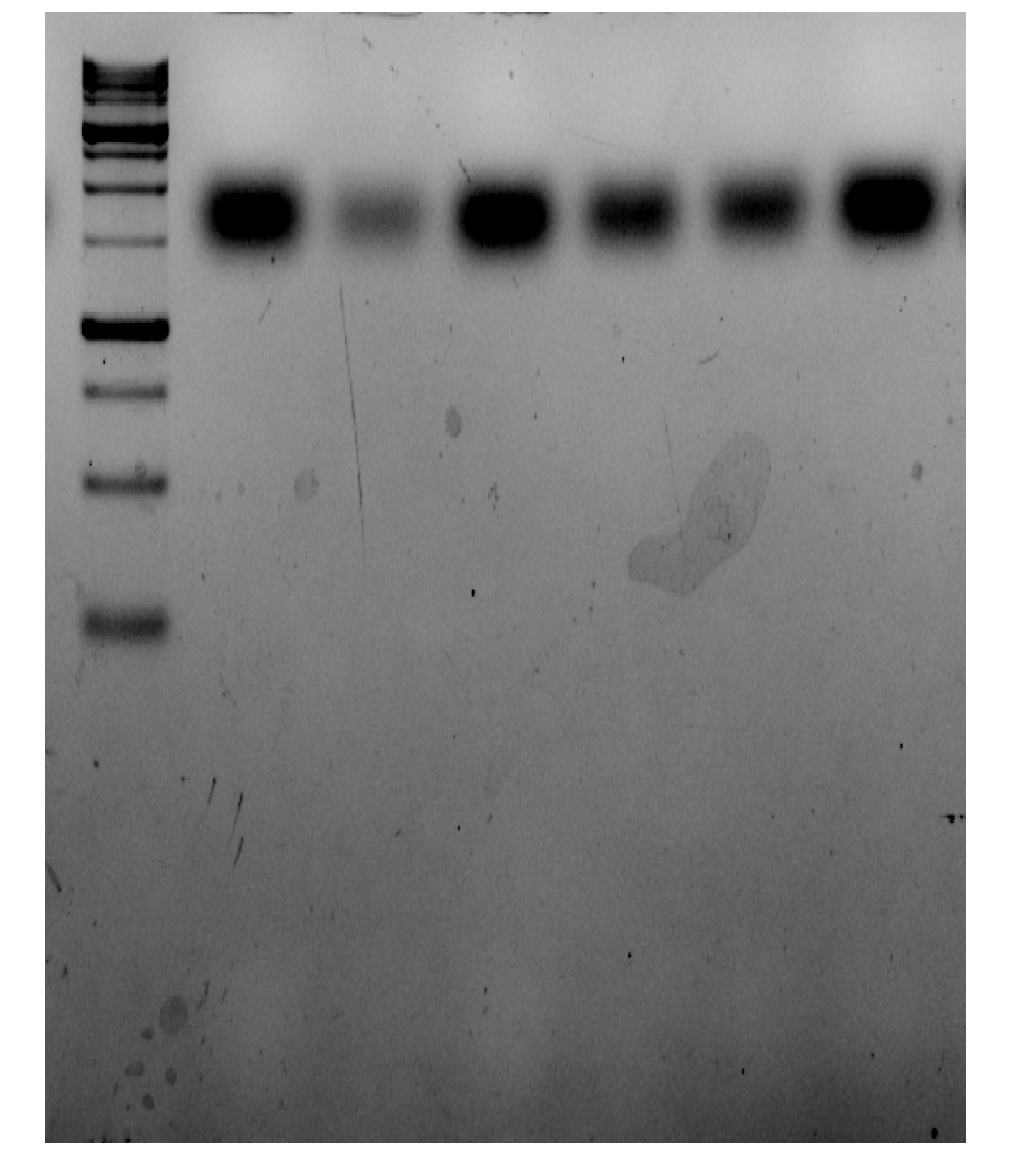

Supplement: Supplementary file 7 — Supplementary file7 (JPG 1146 KB) [file 210_2024_3026_MOESM7_ESM.jpg]

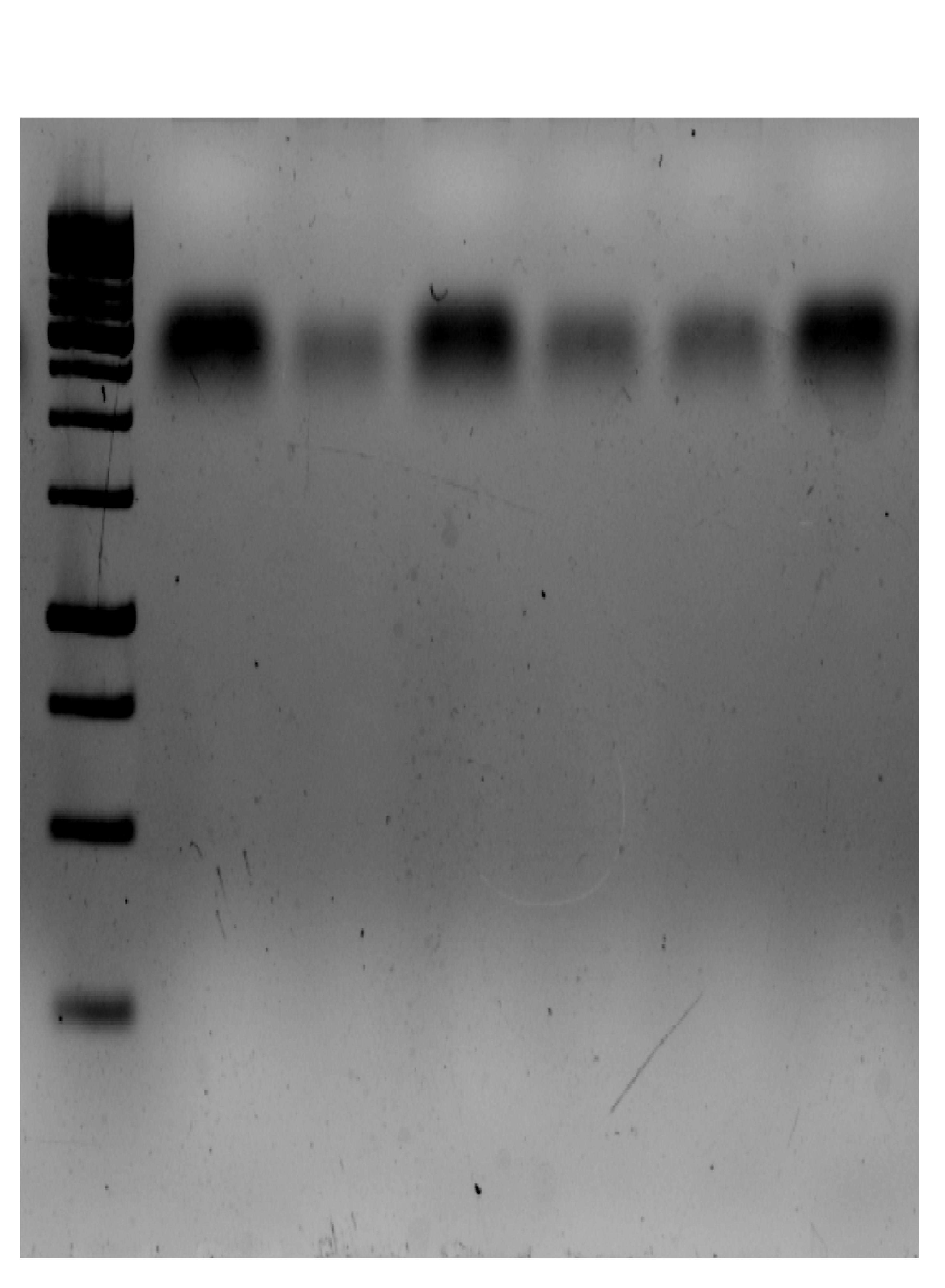

Supplement: Supplementary file 8 — Supplementary file8 (JPG 1023 KB) [file 210_2024_3026_MOESM8_ESM.jpg]

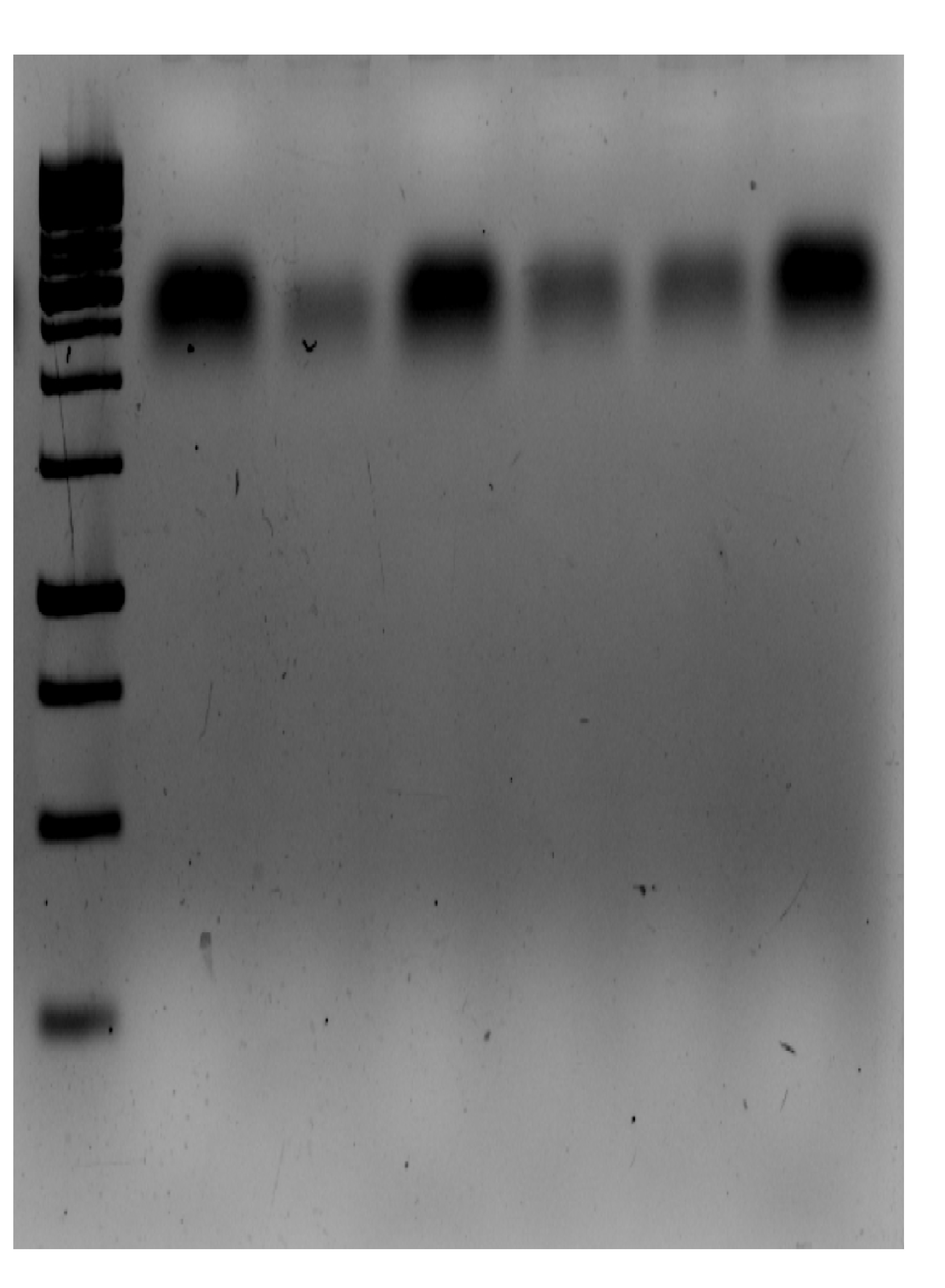

Supplement: Supplementary file 9 — Supplementary file9 (JPG 1058 KB) [file 210_2024_3026_MOESM9_ESM.jpg]

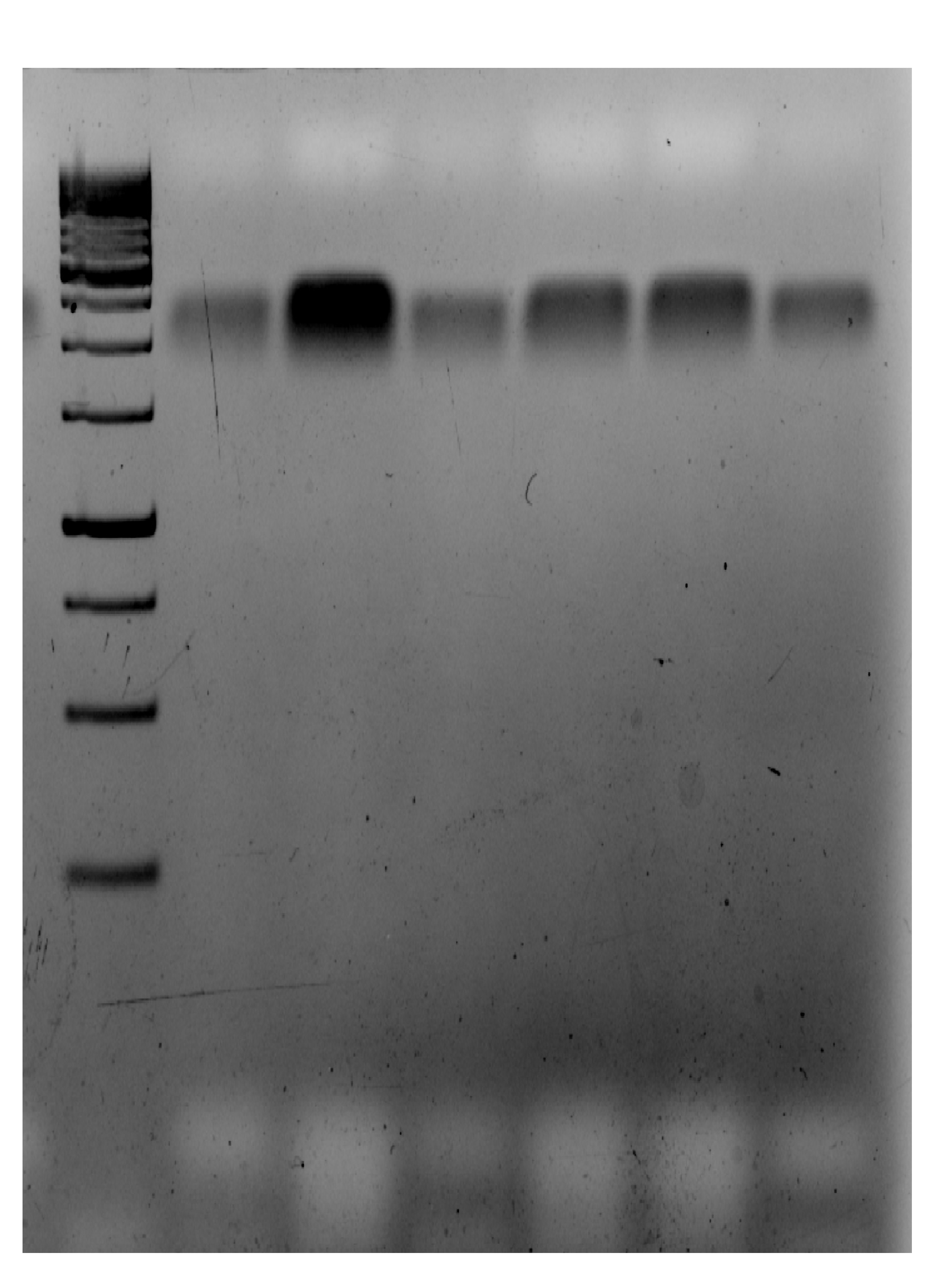

Supplement: Supplementary file 10 — Supplementary file10 (JPG 1298 KB) [file 210_2024_3026_MOESM10_ESM.jpg]

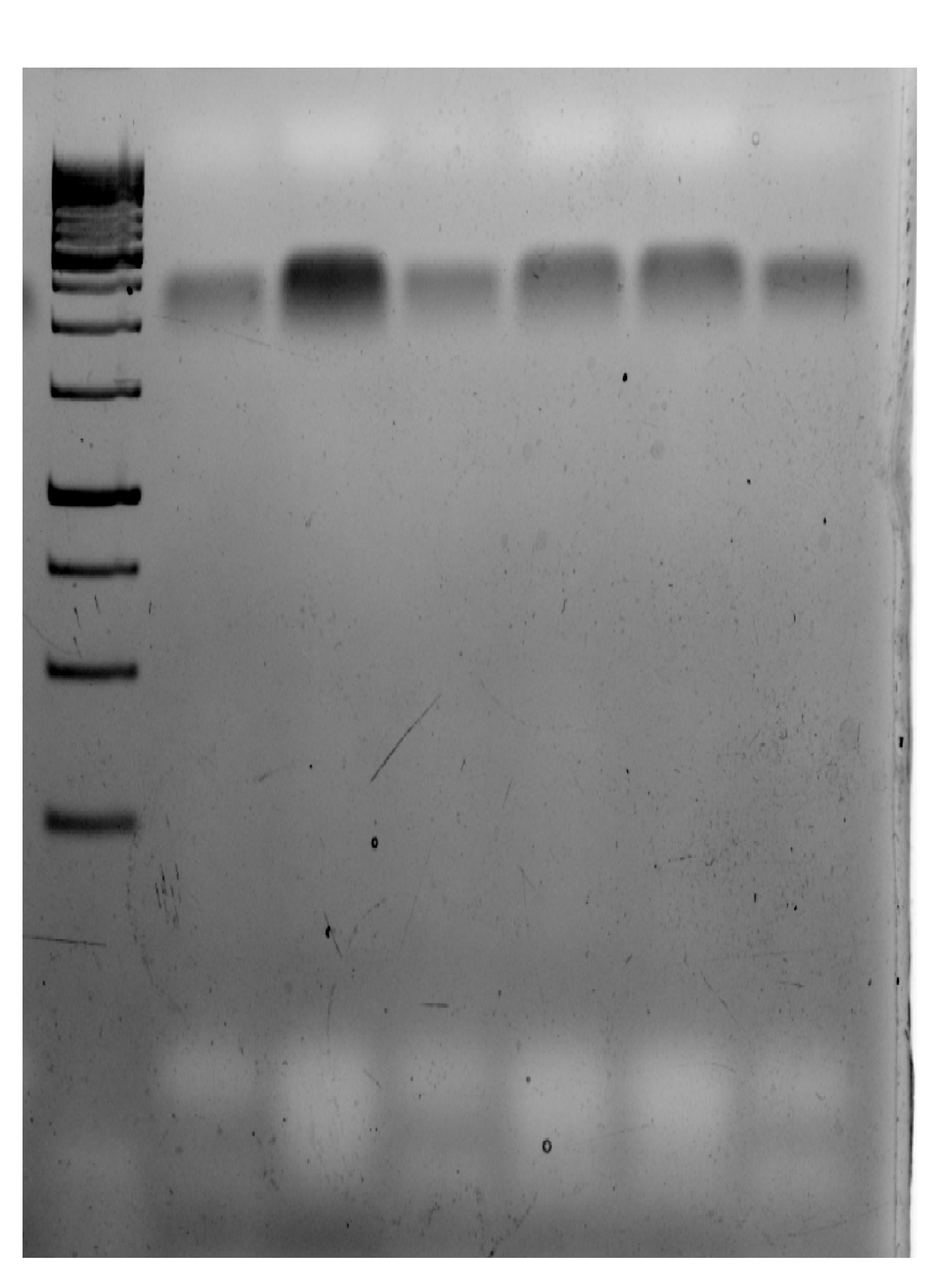

Supplement: Supplementary file 11 — Supplementary file11 (JPG 1319 KB) [file 210_2024_3026_MOESM11_ESM.jpg]

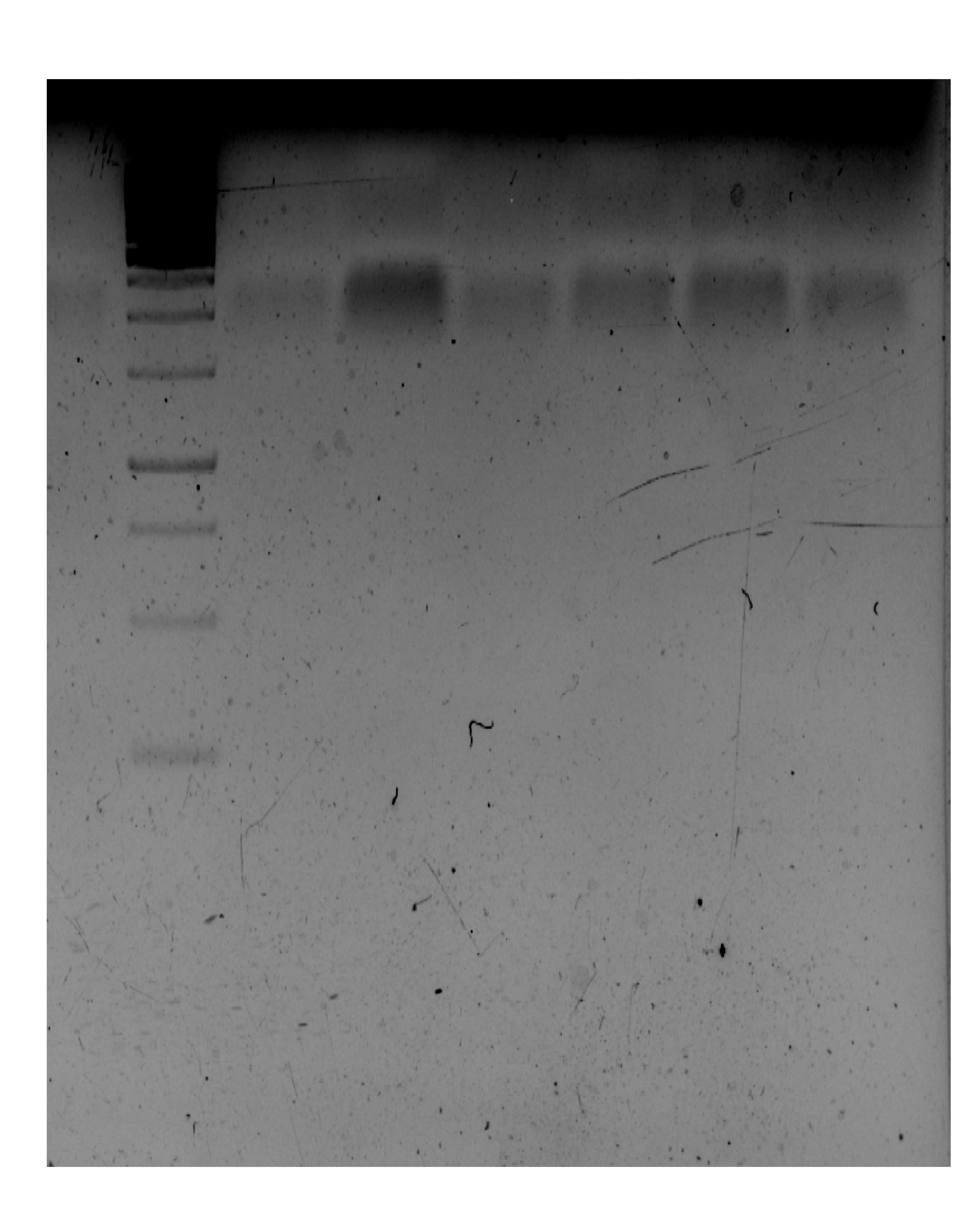

Supplement: Supplementary file 12 — Supplementary file12 (JPG 1177 KB) [file 210_2024_3026_MOESM12_ESM.jpg]

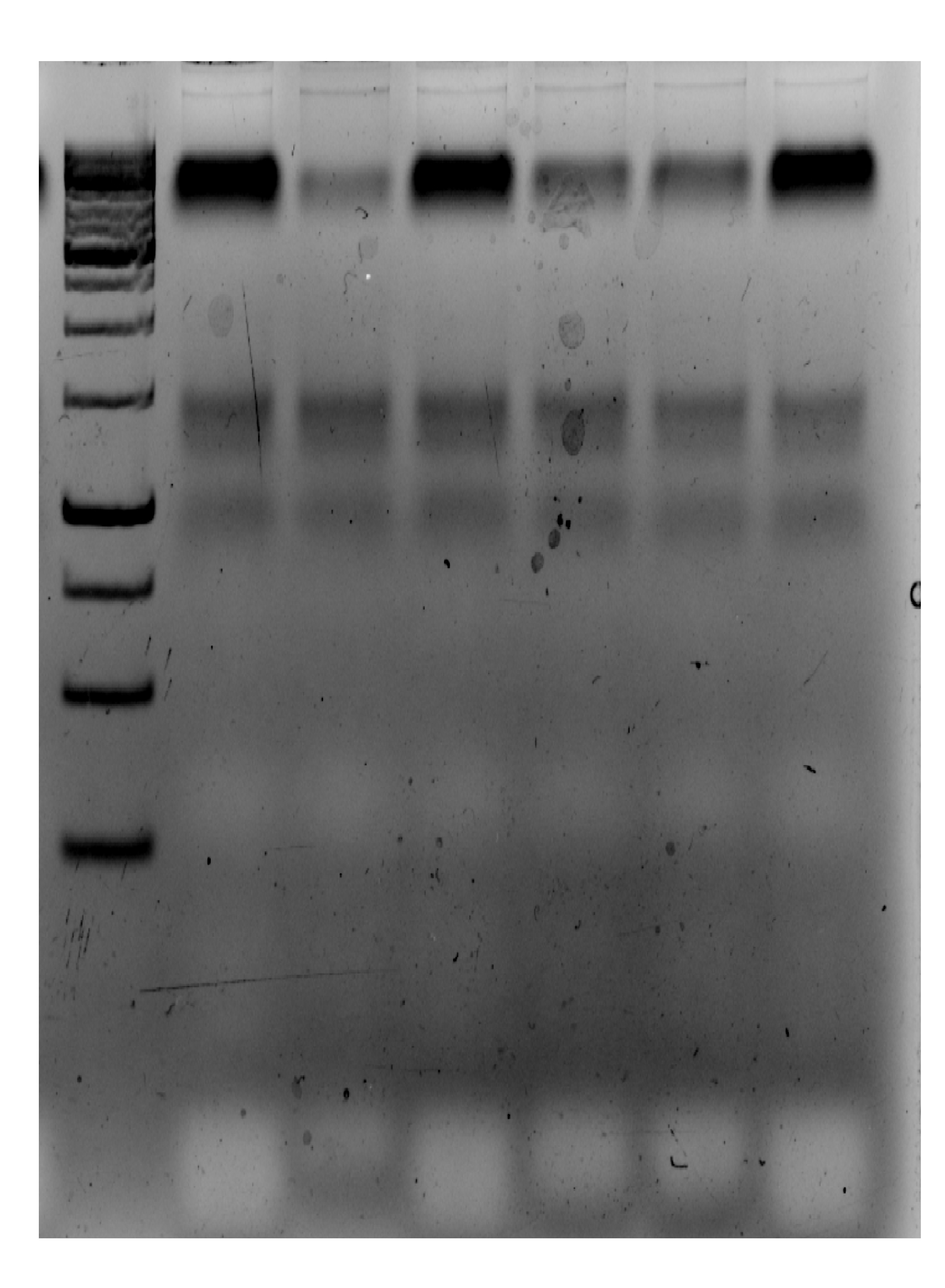

Supplement: Supplementary file 13 — Supplementary file13 (JPG 1366 KB) [file 210_2024_3026_MOESM13_ESM.jpg]

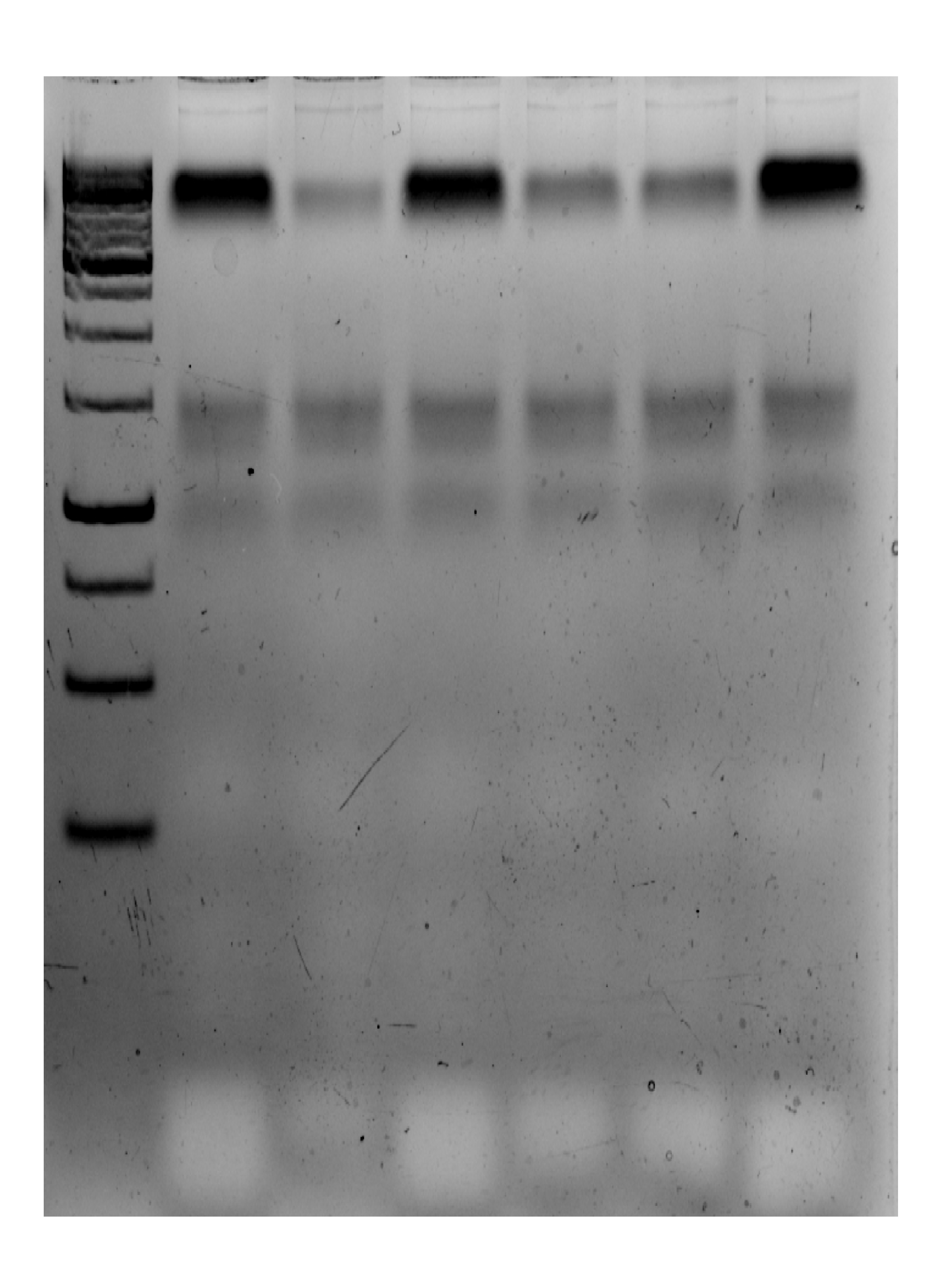

Supplement: Supplementary file 14 — Supplementary file14 (JPG 1275 KB) [file 210_2024_3026_MOESM14_ESM.jpg]

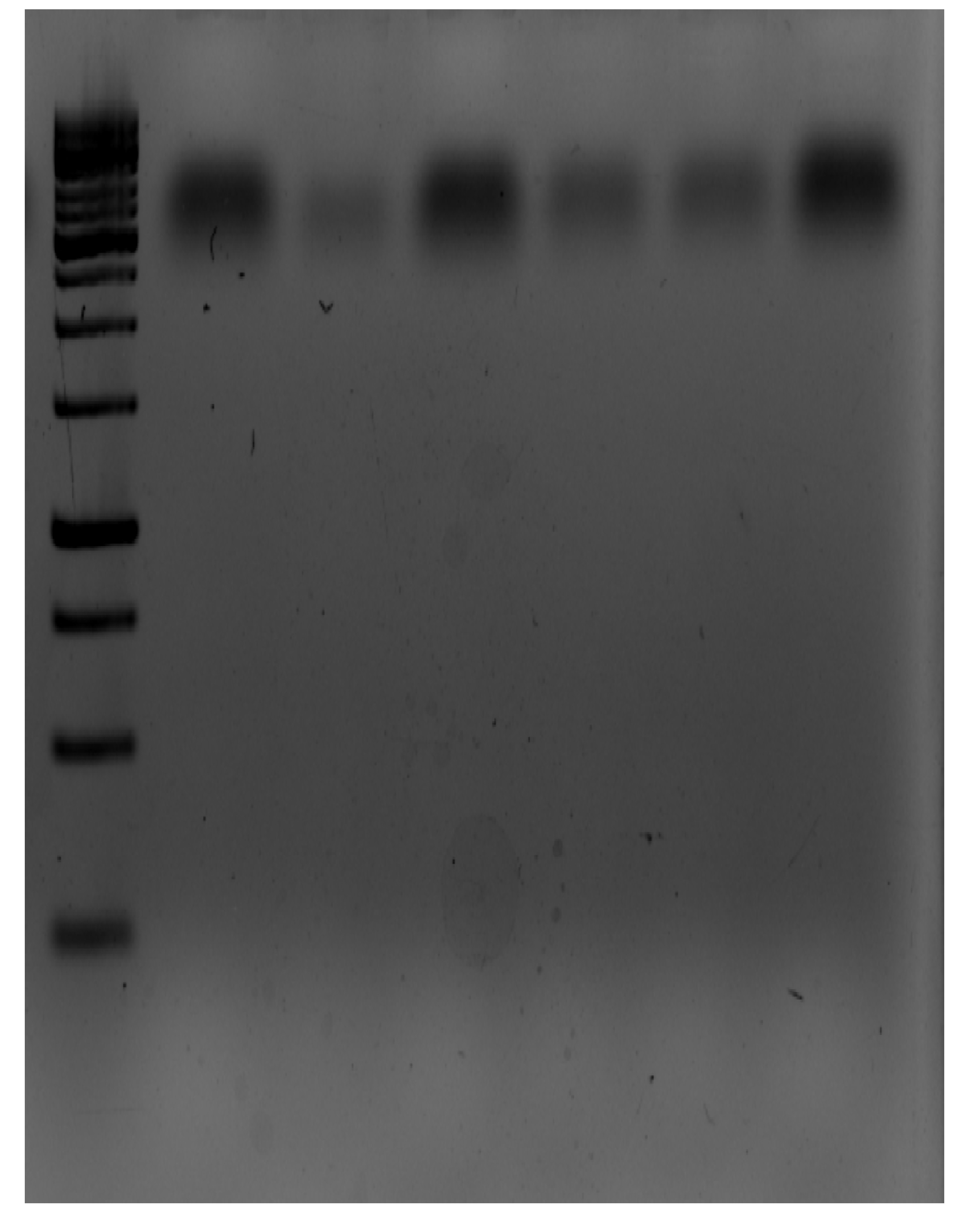

Supplement: Supplementary file 15 — Supplementary file15 (JPG 787 KB) [file 210_2024_3026_MOESM15_ESM.jpg]

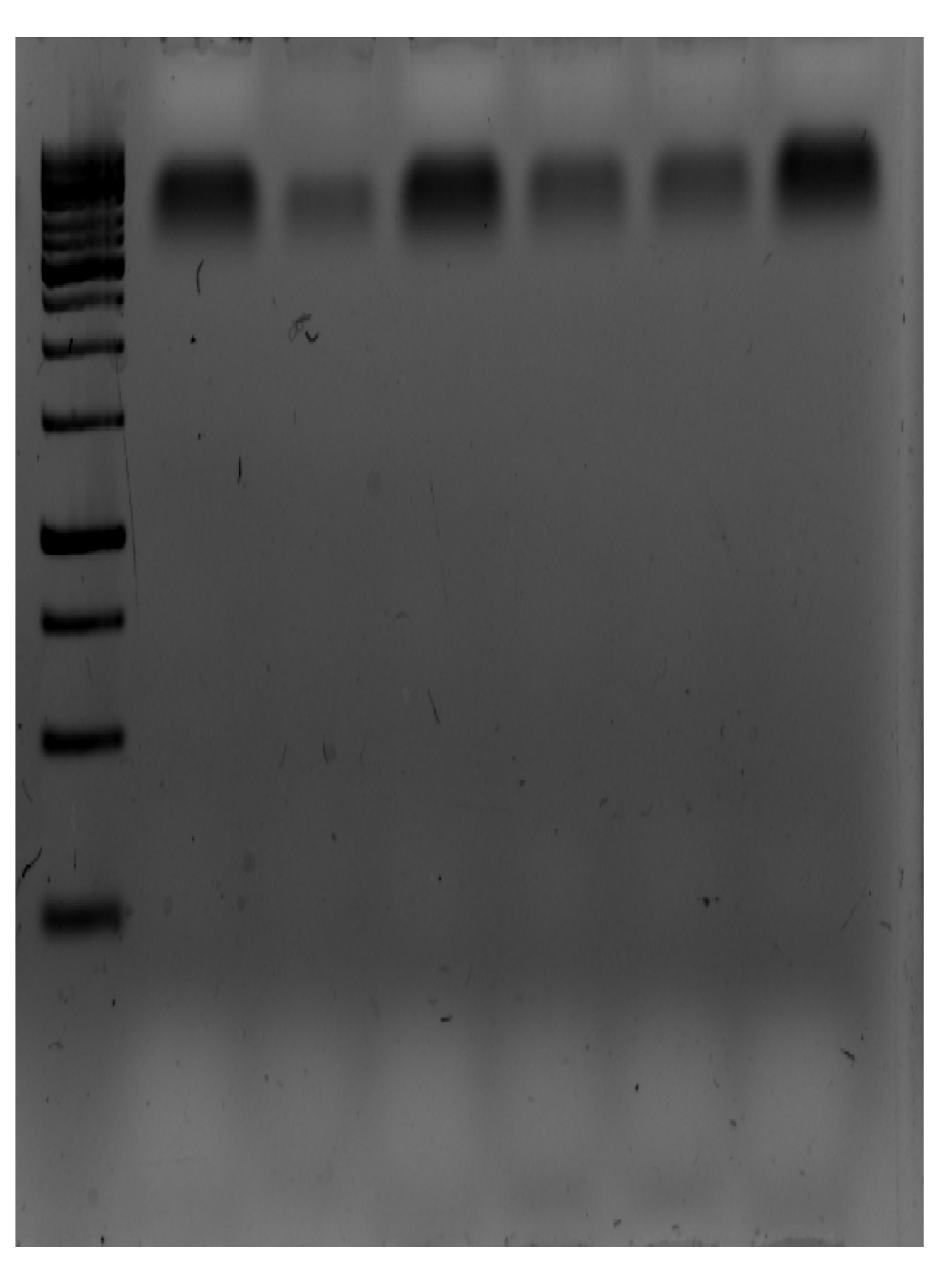

Supplement: Supplementary file 16 — Supplementary file16 (JPG 903 KB) [file 210_2024_3026_MOESM16_ESM.jpg]

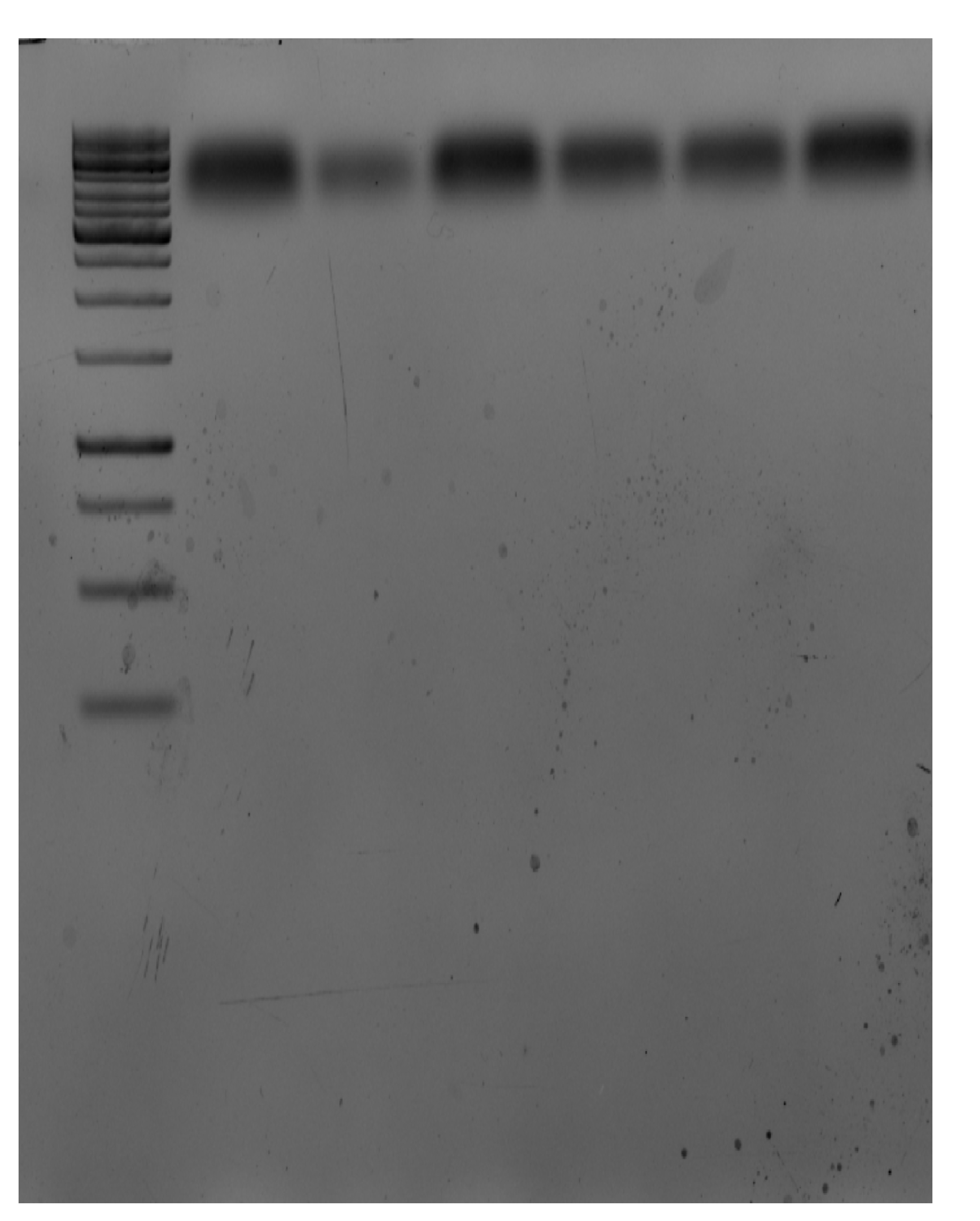

Supplement: Supplementary file 17 — Supplementary file17 (JPG 886 KB) [file 210_2024_3026_MOESM17_ESM.jpg]

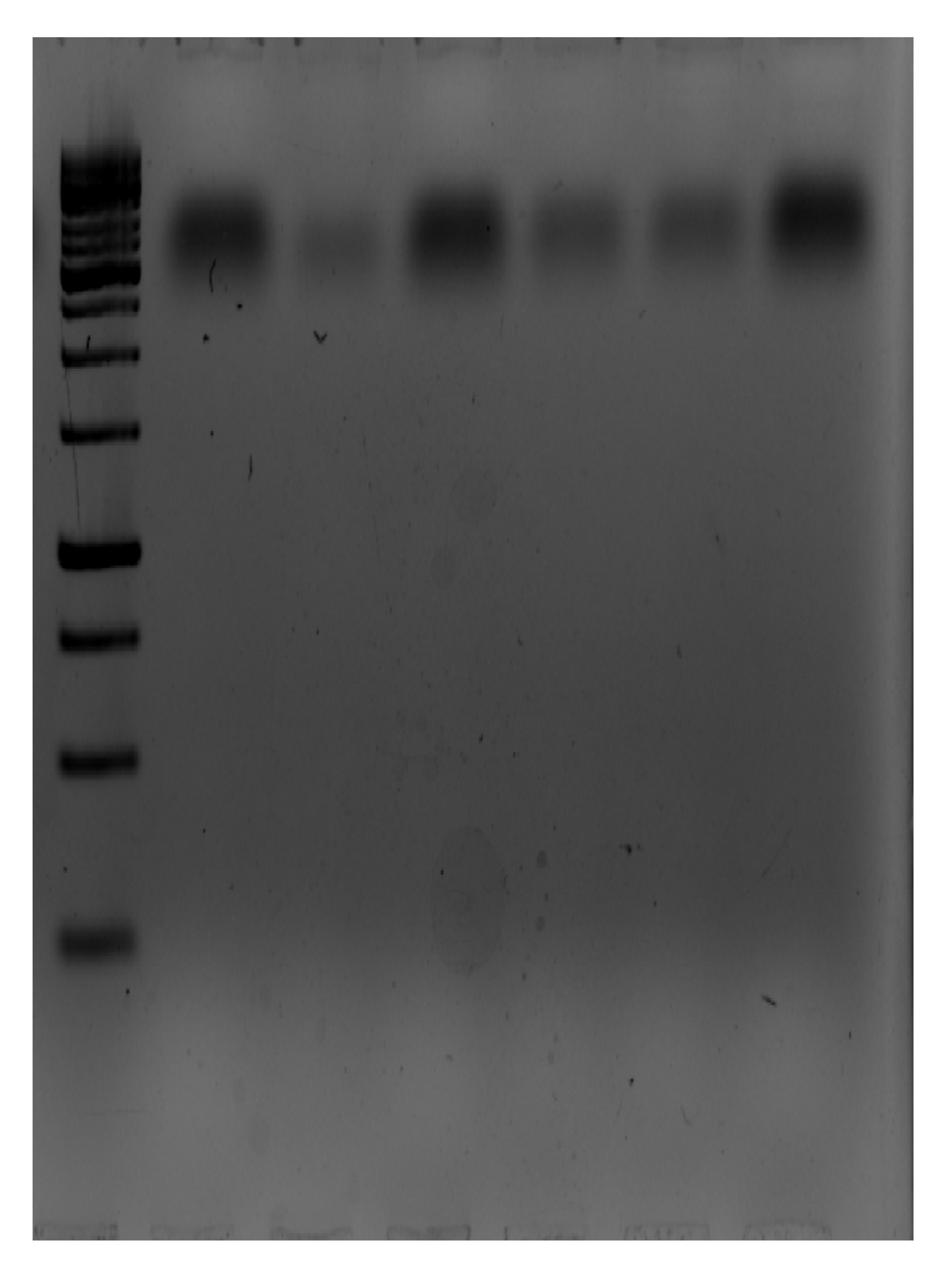

Supplement: Supplementary file 18 — Supplementary file18 (JPG 866 KB) [file 210_2024_3026_MOESM18_ESM.jpg]
